# Exposure to 5G-NR electromagnetic fields affects larval development of *Aedes aegypti* mosquito

**DOI:** 10.1038/s41598-025-32816-y

**Published:** 2025-12-25

**Authors:** Eline De Borre, Charles De Massia, Matthieu N. Boone, Pie Müller, Arno Thielens

**Affiliations:** 1 Department of Information Technology, Ghent University - Imec, Ghent, 9000 Belgium; 2 Swiss Tropical and Public Health Institute, Allschwil, 4123 Switzerland; 3 University of Basel, Basel, 4001 Switzerland; 4 Centre for Xray Tomography (UGCT), Department of Physics and Astronomy, Ghent University, Ghent, 9000 Belgium; 5 Photonics Initiative, Advanced Science and Research Center, The Graduate Center of the City University of New York, New York, 10030 USA

**Keywords:** Yellow fever mosquito, Radio-frequency, Electromagnetic Exposure, Reverberation Chamber, Insect Development, 5G, Applied physics, Ecology, Entomology

## Abstract

Telecommunication networks, including 5G New Radio (5G-NR), emit these fields and consequently expose many insects. To quantify the potential effect of RF-EMF exposure on insects, a study was designed examining the development of the *Aedes aegypti* mosquito, a major vector of dengue and other pathogens, as model organism exposed to RF-EMFs at 3.6 GHz. A custom exposure setup, a reverberation chamber, was designed, built, and characterized. Numerical simulations made it possible to calculate doses received by the larvae during the exposure. Larvae were reared on two feeding regimes, differing in nutritional value, and exposed for 5 days. At an RF exposure level of 46.2 V/m and absorbed power of 1.2 $\upmu$W, a slower development occurred, especially for weakened larvae. At an RF exposure level of 182.6 V/m and 18.7 $\upmu$W absorbed power, dielectric heating changed development timing and adult size.

## Introduction

The yellow fever mosquito, *Aedes aegypti,* is mostly known as an arbovirus vector for pathogens such as yellow fever, dengue fever or Zika fever virus^1^. Mosquitoes are also contributors to pollination and serve as prey for other animals^2,3^. Every environmental impact, positive or negative, is thus of great interest to us as it might influence their control and proliferation.

An underexplored environmental factor that might influence *Ae. aegypti* is exposure to radio-frequency electromagnetic fields (RF-EMFs) emitted by wireless telecommunication networks. These have become a big part of our daily lives and industries, and as a consequence nearly all organisms in our environment are exposed to RF-EMF, in particular *Ae. aegypti*, which prefers humans as a blood source and thus spends significant time in the vicinity of RF-EMF emitting devices of humans. Insects do not interact in the same way with radio-frequency electromagnetic fields (RF-EMFs) as humans and other vertebrates do. A difference in size^4,5^ and tissue^6,7^ will lead to a difference in profile of absorbed electromagnetic power and possibly in biological effects^8^, such as developmental changes. Additionally, flying insects can appear in the vicinity of RF-EMF emitting base stations and can receive relatively high exposures. Despite the growing interest in the effects of EM exposure on insects^8^, today, there are no regulations on the RF-EMF exposure of insects or other invertebrates.

Previous studies have shown that exposure to EMFs could affect the development of different insects^9–11^. In^12^, *Culex pipiens* adults under exposure of 50 Hz showed a decrease in oxidative stress parameters, lower protein and lipid count, lower body weight and even malformations at high field strengths. In^13^, *Cx. pipiens* larvae were treated with pulsed electric fields, and they again showed oxidative stress effects and disruption of proteins. When looking at RF-EMF exposure in particular, the collective behavior of *Ae. aegypti* exposed to a low-powered sweep between 0.01-20 GHz was studied in an inconclusive pilot study^14^. Halim et al.^15^ found an accelerated development of *Ae. aegypti* after RF-EMF exposure of eggs at 900 MHz and 18 GHz. Additionally, more adults emerged at 900 MHz. However, in their horn antenna setup, the exposure level and dose had not been determined. In^16^, the early life stage of *Ae. aegypti* were exposed to 900 MHz and 18 GHz, showing an effect on larval development duration at 18 GHz depending on temperature. However, once again, neither exposure level nor dose was reported, making it impossible to interpret the results and translate them to real life situations. From these limited studies, one cannot draw conclusions on how RF-EMF exposure affects *Ae. aegypti* and its larval development at 3.6 GHz.

Therefore, our goal was to (i) study the development of *Ae. aegypti* mosquitoes when exposed to 3.6 GHz RF-EMFs during the larval stage, a carrier frequency widely used for 5th generation New Radio (5G-NR) technology, and to combine it with (ii) dosimetric numerical simulations and (iii) the characterization of the experimental setup. Exposure of the larval stage was chosen as it is a critical stage in the mosquito life cycle, during which mosquitoes feed and grow. It is known that environmental factors, such as temperature, density, and diet can greatly influence the developmental rate and the survival of mosquitoes^17–19^ and were thus taken into account. We exposed the larvae using a continuous wave at 3.6 GHz, rather than working with a time-modulated signal, such as the ones used in wireless networks, because we want to strictly study the effect of the RF waves. When applying a high RF-EMF, dielectric heating of the larva and the surrounding water can occur^20^, increasing the temperature. Therefore, exposure levels were set that allowed for differentiation between thermal levels. Potential developmental effects were quantified using several parameters for a large sample size (total of 3360) of larvae. The size of the eclosed adult mosquitoes was assessed through wing length measurements as a proxy for the body size, and asymmetry between left and right wing length was measured, both as an indicator for fitness^21,22^. Further, mortality and developmental duration were monitored from larval instar 1 (L1) to pupation and to adult emergence.

To expose many larvae simultaneously and uniformly in vivo, a reverberation chamber (RC) was customized. The RC is an electrically large overmoded cavity with a high quality factor (Q-factor), generating a statistically uniform field by employing rotating stirrers^23^. Exposure devices often come with large uncertainties and characterization is not always done well. Here, the RC characterization was done as in^24,25^, accompanied by an extra metric to evaluate the EMF uniformity further. An RC has been used for in vivo experiments on vertebrates before^26^, however not for insects.

To correctly interpret the results of the exposure experiments, the dose received by the larvae during the experiment was determined. Therefore, finite-difference time-domain (FDTD) simulations were performed, similar to the ones in^4,27^. To this aim, a new 3D model was created with micro-computed tomography ($\upmu$-CT) scanning for the *Ae. aegypti* larva simulations. In^28^, simulations with adult *Ae. aegypti* showed a frequency dependency of the absorbed EM power. Simulations using FDTD have often been used for dosimetric studies for both in vivo^29–31^ and in vitro^25^ experiments with an RC as exposure setup.

The aim of this study is to contribute to a comprehensive understanding of the impact of RF-EMF on insects. *Ae. aegypti* was used as a model organism to study the developmental effects of RF-EMF exposure of insects. The studied effects and related RF-EMF dose can serve as an input into possible guidelines and policy making regarding RF-EMF exposure of insects.

## Materials and methods

Before conducting the EM exposure experiments, a specialized reverberation chamber (RC) was constructed and characterized to ensure controlled conditions. Further, numerical simulations were performed to model the RF-EMF exposure of the larvae under experimental settings, providing the dosimetric data necessary to interpret the exposure experiment outcomes.

### RC materials and design

**Fig. 1:**
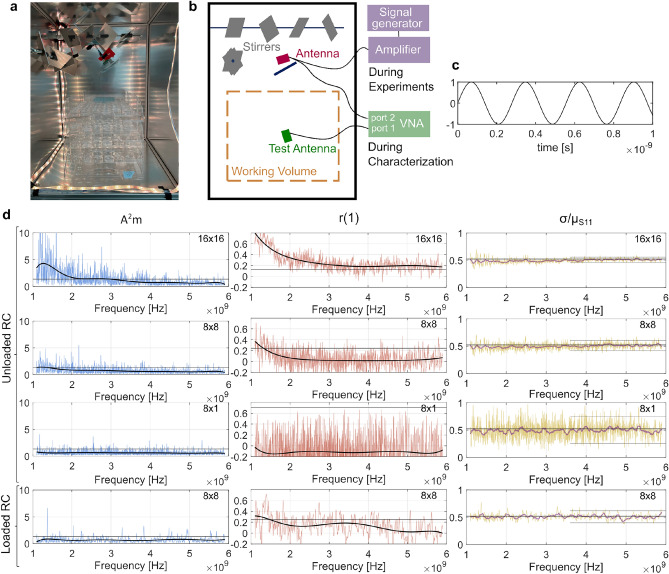
The electromagnetic exposure setup and its characterization. (**a**) Picture of the inside of the reverberation chamber (RC). (**b**) Schematic front view of the RC with setup during characterization and during exposure experiments. (**c**) Continuous sinusoidal wave of frequency 3.6 GHz. (**d**) The modified AD-statistic ($A^{2}_{m}$), first order correlation factor (*r*(1)), and the ratio of the standard deviation and the mean of $| S_{11}^{sti} |$ ($\sigma /\mu$) or coefficient of variation (*CV*) of the N (stirrer positions) measurements for the unloaded RC with stirrer positions uniformly divided over $360^{\circ }$ and for the loaded RC with Petri dishes filled with water. The amount of stirrer positions (N = stirrer 1 positions $\times$ stirrer 2 positions), is indicated in the right top corner of each subfigure. The polynomial fits (thick black line) of $A^{2}_{m}$ and *r*(1) are of order 8 and 6, respectively. The thick purple line for *CV* shows the moving mean over 100 points (or 100 MHz). The threshold for $A^{2}_{m}$ is at 1.341, while the one for *r*(1) is at 0.123, 0.246 and 0.707 for N = 256, 64 and 8, respectively. The horizontal black line in the *CV* graphs is at 0.523 and the grey lines in the *CV* plots show the 95% interval calculated for frequencies above 3.6 GHz.

A reverberation chamber (RC) was built, see Fig. 1. The RC with dimensions 466 mm $\times$ 436 mm $\times$ 600 mm was constructed out of 1 mm thick aluminum plates. The dimensions were chosen so that the RC was certainly functional at 3.6 GHz (calculations in Supplementary Information). The lowest usable frequency, the frequency at which there are approximately 100 modes, is theoretically estimated to be 1.411 GHz^32^. Two aluminum stirrers were installed inside the RC (to change the EMF in the RC), consisting of four squares with sides of 86 mm placed at different angles with respect to the rotation axis. Two plates at each stirrer were placed at 45$^{\circ }$ to the rotation axis and at 90$^{\circ }$ to each other. One plate had two of its sides, and the other its diagonal, perpendicular to the rotation axis. The other two plates of the stirrer were similar but made an angle of 30$^{\circ }$ with the rotation axis and of 120$^{\circ }$ with each other. The volume of the smallest cylinder containing a stirrer was determined for both stirrers. The total stirrer volume as a percentage of the chamber volume was 4.3%^33,34^. During the exposure measurements, 8$\times$8 stirring positions were used. For each of the 8 positions of stirrer one, the other stirrer took 8 positions, all uniformly distributed over 360$^{\circ }$. The stirrers moved at a speed of 7.32 RPM and stirrer positions were held fixed for 5 seconds. A half wavelength dipole antenna was used as a transmitter in the RC, at a frequency of 3.6 GHz. Another aluminum plate, of the same dimensions as the stirrer plates, was placed between the dipole and working volume and prevented direct exposure. In this way, the antenna’s radiation was directed more towards the stirrers and not the insects. Antenna and working volume were kept at least $\lambda / 4$ from the sides of the RC, the stirrers and the mentioned plate. The antenna was connected to an amplifier (Model 5S1G4, AR RF/Microwave Instrumentation), which in turn was connected to a signal generator (BSPG, Aaronia AG, Strickscheid, Germany) generating a sinusoidal continuous wave as shown in Fig. 1.c. Further characterization and calculation for the antenna and RC are given in the Supplementary Information, Fig. S1-3.

### RC measurements

To ensure that the new RC was functional at 3.6 GHz, the $S_{11}$ parameter was measured for the RC using a Vector Network Analyzer (VNA ZNB20, Rhode & Schwarz, Munich, Germany) in the RC. An example of a $S_{11}$ measurment is given in supplementary Fig. S1. The VNA was connected to a broadband antenna (Model XPO2V-0.8-6.0-GF/1441, Chelton Limited, Suffolk, UK) placed inside the working volume of the unloaded RC (Test Antenna in Fig. 1) or placed next to the filled Petri dishes in the case of the loaded RC. The measurements were done between 1.1 GHz and 5.9 GHz with steps of 1 MHz, repeated for every 16$\times$16 stirrer positions. Stirrer positions 8$\times$8 and 8$\times$1 were taken as a subset from the original data with equally distributed angles between the positions per stirrer over 360$^{\circ }$. For the measurements on a loaded chamber, Petri dishes were filled with water and placed inside the RC just like in the exposure experiment.

### RC calculations and statistics

The RC is small compared to the measurement equipment at hand, and investigating field uniformity in the RC using Cartesian electric field values^35,36^ was not possible. Instead, the method of^24^ was used, where $S_{11,i}^{st} = S_{11,i}^{RC} - \langle S_{11}^{RC} \rangle$ is investigated. Here, the $S_{11,i}^{RC}$ is the measured $S_{11}$ for position *i* of the N positions, $\langle S_{11}^{RC} \rangle$ is the average over all N positions, and $S_{11,i}^{st}$ is the $S_{11}$ contributed by the stirrers in stirring position *i*. For the EMFs to be uniform, the $| S_{11}^{st} |$ needs to be Rayleigh distributed. The Anderson-Darling (AD) goodness-of-fit (GOF) test, was used to test the N values of $| S_{11,i}^{st} |$ against the Rayleigh distribution for each frequency. The modified AD statistic $A^{2}_{m}=A^{2}(1+0.6/N)$ was calculated, with $A^{2}$ the AD statistic. For $A^{2}_{m} > 1.341$, the data fit the Rayleigh distribution with a significance level $\alpha$ of 5%.^24,37^.

1 $$\begin{aligned} A^2=-N-\frac{\sum ^{N}_{i=1}(2i-1)[lnF(x_i)+ln(1-F(X_{N+1-i}))]}{N} \end{aligned}$$

With *F*(*x*) the cumulative distribution function for the Rayleigh distribution and $x_i$ the ordered experimental value with rank *i*.

As a second step in characterizing the RC in^24^, the independence of the measured samples over the different stirrer positions was examined. The first-order auto-correlation coefficient *r*(1) was calculated for all frequencies.^24,38^

2 $$\begin{aligned} r(1)=\frac{\sum ^{N}_{k=1}(x(k)-\langle x \rangle )(x(k+1)-\langle x \rangle )}{\sum ^{N}_{k=1}(x(k)-\langle x \rangle )^2} \end{aligned}$$

With *x* the N experimental values ($| S_{11}^{st} |$) per frequency. Depending on the amount of stirrer positions, *r*(1) needs to be smaller than a threshold $t_2$ for the stirrer positions to be considered independent. The $t_2$ was 0.707 for N=8, 0.246 for N=64 and 0.123 for N=256^39^.

A Rayleigh distribution’s ratio of the standard deviation to the mean $\sigma / \mu$ for the measured N values of $S_{11}^{sti}$ at every frequency, also called the coefficient of variation *CV*, was approximately 0.523^40^. Hence, in the characterization of the RC, it was checked whether this ratio is close to this value above a certain frequency (here 2.74 GHz), the 95% interval was calculated on *CV* (*CV* ± $2 \times$ the standard deviation of *CV*).

### 3D models

The geometric models of the larvae were spheroids with the same length as the full body length and the same width as the width of the head of the different instars in^41^. This resulted in volumes close to the ones of the anatomical model, and thus usable for these simulations^5^. Dimensions of the model are shown in Table S1. The anatomical model for an *Ae. aegypti* larva at instar 4 (L4) is a reconstruction from micro CT-scans. The larva originated from the same strain as used for the experiments. The earlier instars (L1-3) were too fragile, and their anatomical models were rescaled from the scanned L4 with the length of the different instars according to^41^. Critical point drying was used to prepare the samples for micro-CT-scans and prevent them from collapsing, as done in^42^. For this drying, the samples needed to be dehydrated by increasing ethanol concentrations first, the larvae were already preserved at 70 % ethanol before dehydration.

Micro CT scanning was performed at the Ghent University Centre for X-ray Tomography (UGCT; www.ugct.ugent.be) using the TESCAN CoreTOM scanner (TESCAN, Brno, Czech Republic). A tube voltage of 70 kVp was used, and 3201 radiographic images were acquired, at 1500 ms exposure time per image, during a 360$^{\circ }$ rotation of the samples. The raw data were reconstructed into an image stack representing the 3D volume at a voxel size of 3$^{3}$ $\upmu$m$^{3}$ due to the geometrical magnification used during scanning. The image data stack was loaded into VGStudio Max (Volume Graphics, Heidelberg, Germany), a growing area function was used and based on the grey values the larva was selected. An STL (stereolithography) was extracted from the 3D volume.

### Numerical simulations

Numerical simulations were done using Finite Difference Time Domain (FDTD) in Sim4Life (ZMT, Zurich, Switzerland). From these simulations, the whole-body power absorption $P_{abs}$ was computed for the larvae:

3 $$\begin{aligned} P_{abs} = \int \sigma \times | \vec {E}_{int} | ^{2} \cdot \; dV, \end{aligned}$$

With $\sigma$ the conductivity, $| \vec {E}_{int} |$ the root mean square (RMS) internal electric field strength and *V* the volume. The $P_{abs}$ scales with the square root of the incident electric field strength ($E_{int}$) as shown in Supplementary Information^27^, and thus an RMS $E_{int}$ of 1 V/m was chosen for the simulations for ease.

In the working volume of the RC, the incidence and polarization of the EMF is considered random^43^. The exposed sample in the working volume can then be simulated using plane waves^30,31^. A set of twelve plane waves was chosen^44^ that is expected to result in values of $P_{abs}$ that span all possible $P_{abs}$ the larva could experience in free space at that frequency and intensity. These incident plane waves (incidence and polarization) were orientated along the main axes of the spheroid model in the horizontal position. The $P_{abs}$ was then taken as the mean of the 12 simulations. The minimum and maximum $P_{abs}$ are also given.

The aquatic larva was placed in water (depth of 9.15 mm) in a Petri dish (polystyrene) with a diameter of 3.5 cm. The dielectric properties of water and polystyrene came from the Sim4Life database. The dielectric properties of the larva were taken as 90% adult mosquito from^28^ and 10% water. This choice was made as it can be seen that in earlier life stages of insects (certainly aquatic), a higher water content is present^45^. The dielectric properties used are given in Tables S2. The larva models were spatially discretized in cubic voxels of 0.05 mm and the water and Petri dish in voxels of 0.3 mm. These voxels are < 1/10 of the wavelength in the medium^46^.

**Fig. 2:**
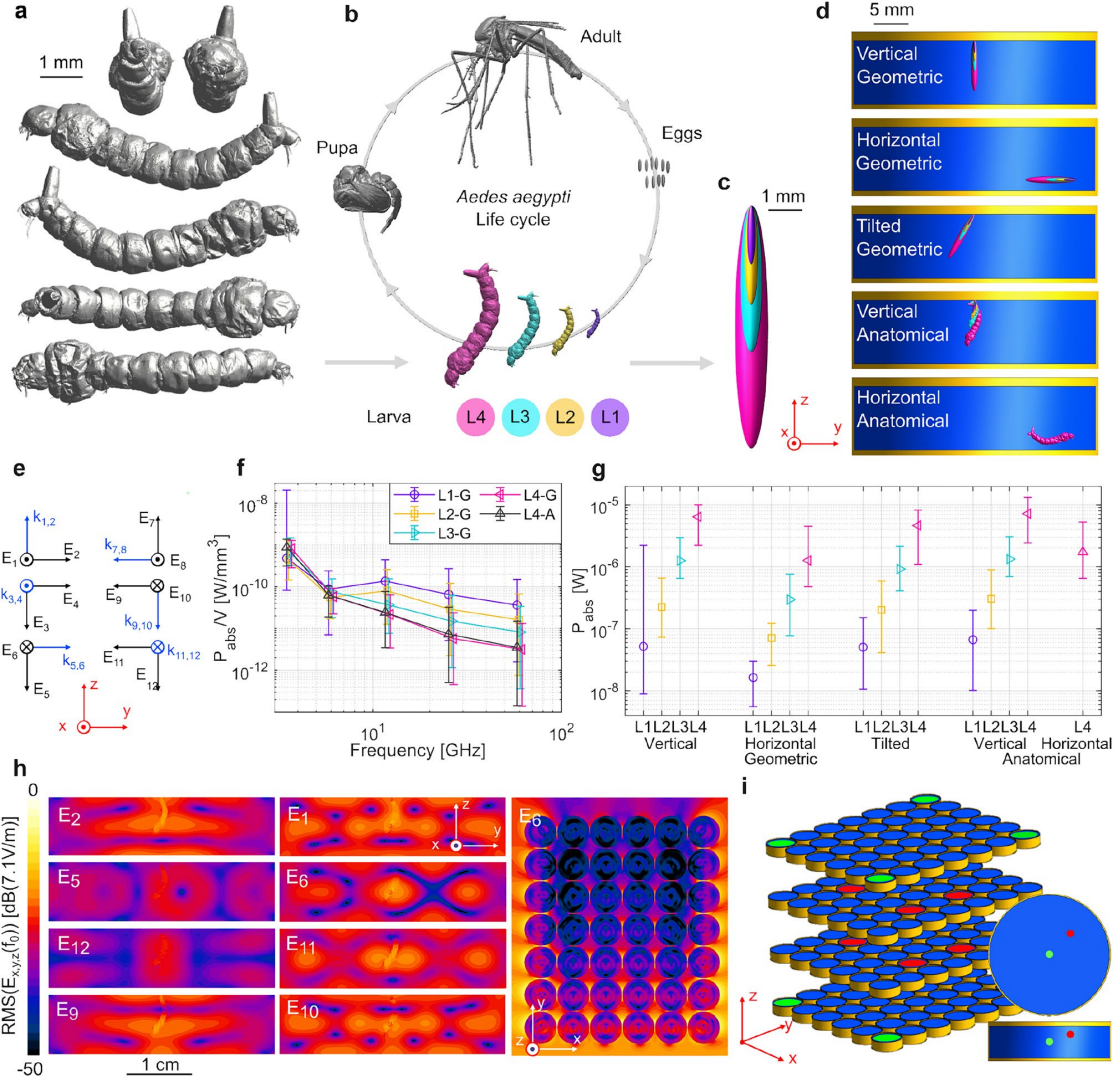
Simulation configuration and results of electromagnetic (EM) exposure on *Aedes aegypti* larva. (**a**) Front, back, left, right, top and bottom view of Anatomical 3D model of larval instar 4, created from micro-CT-scan. (**b**) Life cycle of *Ae. aegypti* using anatomical 3D models, with focus on the four larval instars 1-4: L1-L4. (**c**) Images of geometric 3D model of the four instars, the colors of instars correspond to those of the anatomical models. (**d**) Side view of three positions of the geometric model and two positions of the anatomical model in a Petri dish with diameter 3.5 cm (yellow: polystyrene, blue: water). (**e**) The twelve EM plane waves used in the simulations. (**f**) The simulated $P_{abs}$ normalized to volume of the 3D model in function of frequency. (G: geometric and A: anatomical model) for an incident RMS Electric field strength of 1 V/m. The mean $P_{abs}$ is given for the twelve simulations (plane waves of subfigure (e)), the whiskers show the minimum and maximum. (**g**) The simulated absorbed EM power ($P_{abs}$) of the different larval instars, positions and 3D models for an incident RMS Electric field strength of 46.2 V/m. The mean $P_{abs}$ is given for the twelve simulations, the whiskers show the minimum and maximum. (**h**) Spatial distribution of the RMS Electric field strength for an incident field strength of 1 V/m for the side view of a Petri dish containing water and the anatomical L4 in vertical position, and a top view of the second layer from the top of the Petri dish matrix near the water surface. The plane wave of the simulation is indicated in white. (**i**) 3D model of a matrix of Petri dishes as placed in the reverberation chamber. The green and red water indicate the Petri dishes with most and least EM power absorption, respectively. The green and red points in the Petri dish indicate the points in the Petri dish experiencing the highest and lowest Electric field strength, respectively.

The different instars (L1-L4) exhibit very different volumes, simulations for all four were done on both the geometric and the anatomical model at 3.6 GHz. Different positions were also considered, as seen in Fig. 2d, horizontal and vertical for the anatomical model, for the geometric model also a tilted position of 30$^{\circ }$ compared to the vertical position was added. Geometric models required less simulations due to their symmetry, so the vertical geometric model was used for frequency dependency and as a basis for simulation uncertainties. Uncertainties that were studied are given in the Supplementary Information, Table S3. The uncertainties involved the water level, the dielectric properties of the larva and the water and simulation settings such as voxel size, simulated periods and set of incident plane waves. The water level could vary during exposure experiments and the dielectric properties were not measured, giving rise to uncertainties. The horizontal position of the four geometrical instars, and of the L4 anatomical instar were also considered at other frequencies. For the frequencies 3.6 GHz, 6 GHz, 12 GHz, 26 GHz and 60 GHz, the duration of the simulations were 10, 15, 15, 15, and 21 periods, respectively.

Simulations were also done on the matrix of 168 Petri dishes as they would appear in the RC. The spacing was 6.4 cm between the trays and 0.1 mm between the Petri dishes in the trays. To limit the simulation time, no larvae were used and only the water was considered. Apart from the twelve plane waves given in Fig. 2.e., extra simulations were done to account for shadowing effects as to cover more angles in the sphere around the sample. A total of 26 incidence directions $\times$ 2 polarizations were considered; however, due to symmetry, it was sufficient to run 22 simulations. The plane waves of these extra simulations had their incidence at 45$^{\circ }$ or 135$^{\circ }$ zenith angle, and at every 45$^{\circ }$ interval azimuth angle (see axes in Fig. 2) or 90$^{\circ }$ zenith angle and every 45$^{\circ }$ interval azimuth angle that was not already part of the original 12 plane waves.

### Exposure assessment

The electric field strengths (*E*) in and around the RC were measured using a broadband probe (PMM EP-408 DB, with the PMM 8053b, Narda Safety Test Solutions). Every measurement was a 1-minute average of the RMS of the 3 Cartesian electric fields with the device inside the RC, thus also slightly influencing the field. The highest possible generated field with our equipment was used for High Exposure (HE), this was measured over 69 points for an unloaded chamber and for 305 points with the probe next to the water filled Petri dishes.The same was done for a lower exposure LE (108 measurements unloaded, 574 loaded). In the control experiments, 10 measurements were done on the unloaded chamber, showing an *E* lower than 0.8 V/m. The *E* of the loaded chamber in the control situation was below the detection limit of the device. The *E* around the RC never exceeded the International Commission on Non-Ionizing Radiation Protection (ICNIRP) limits^47^.

Exposure values in electric field strength (E, [V/m]) can be transformed into power densities (S, [W/m$^2$]) using $S=E^2/377~\Omega$.

### Insect

*Aedes aegypti* larvae of the Rockefeller laboratory strain reared at the Swiss Tropical and Public Health Institute (Swiss TPH, Allschwil, Switzerland) were used. Each experiment used 168 insects, thus a total of 3864 were used (4 experiment types x 5 repetitions, + 3 extra in the low temperature control), of which 3360 were used in the results. All insects of LE, HE and LC 0-2 came from the same generation G1. HC 1-4 came from the next generation G2, while HC5, LC 3-5 came of the G3, and LC 6-7 came from G4.

### Experimental protocol

Eggs on a filter paper were hydrated 24 hours before the start of an experiment. Larvae of instar L1 were taken and transferred to separate Petri dishes of diameter 35 mm and height 10 mm to eliminate competition for food^48^. The Petri dishes were filled with ± 1 cm of tap water with Tetra AquaSafe (5mL/10L) suitable for the larvae. Four trays were filled with 6x7 Petri dishes each.

Half of the Petri dishes were fed with a sub-optimal milk (M) feeding regime to weaken the larvae. The other half were fed with ground Tetramin (T) ad libitum^49,50^. The milk was prepared as 2 ml skimmed milk powder in 28 ml water. One droplet of milk solution was given to each Petri dish with a pipette. See also preliminary experiments in Supplementary Information, Fig. S10-11 and Table S4-7 for determining diet and container. The two diets were applied in a checkerboard configuration in each tray (or level) to prevent the placement from influencing the result.

The four trays were then placed in the RC (day 0). In the RC, a LED light strip was present to simulate a 12 light/dark cycle, trays were placed during the light cycle. The RC itself was placed in a thermostatic cabinet (Aqualytic Thermostatic Cabinet Tc 256g 438235), where temperature could be controlled. Temperature and humidity were monitored (Tinytag View 2, TV-4050). The humidity was recorded as 80±10% and the ambient temperature as 28.1 ± 0.2$^\circ$C for all experiments except for HC for which 30.8 ± 0.4$^\circ$C was recorded. The temperature of the water in the Petri dishes and RF-EMF strength depended on the experiment, 4 experimental conditions were considered. Low Temperature Control (LC) with 28.23 ± 0.63$^\circ$C, RF off. Low EM Exposure (LE) with 28.16 ± 0.19$^\circ$C and electric field strength of 58.19 ± 10.72 V/m for the unloaded chamber and 46.18 ± 7.69 V/m for the loaded chamber. High Temperature Control (HC) with 30.33 ± 0.11$^\circ$C, RF off. High EM Exposure (HE) with 29.95 ± 0.70$^\circ$C and electric field strength of 278.91 ± 39.36 V/m for unloaded chamber and 182.63 ± 35.13 V/m for the loaded chamber. With the errors being the standard deviation. Water temperature was measured immediately after exposure. At LE, the RMS electric field strength was below the ICNIRP reference level for the general public (61.4 V/m for plane waves). At HE that reference level was exceeded. Number of temperature measurements of the water: 38 for HE, 84 for LC, 96 for HC, 7 for LE.

Every day at the same time ±2 hours, during the light phase of the cycle, the larvae were fed, while the developmental stages of the insect and deaths were logged. At day 5, the insects were taken out of the RC and further reared in a separate climate chamber at 28±0.2$^{\circ }$C and humidity 80±0.5% (Memmert ICH750Leco), at the same light/dark cycle with light at 30%. Feeding and logging continued until adult emergence. Every experimental condition was repeated 5 times. Except the LE experiments, which was repeated 8 times, of which 5 were selected for analysis. The 3 discarded runs had many deaths early in the experiment which were believed to be caused by an infection. The runs were compared in Supplementary Information Table S14.

Wing length was measured from the axillary incision (alular notch) to the apex of the wing, excluding the fringe^21^. In all runs of HC, and HE3-5, LC 2-4 and LE 3-5, wing length of 10 males and 10 females were measured for both wings (Supplementary Information, see Table S15). Sex ratio’s were also determined, see Supplementary Table S18.

### Experimental statistics

Timing of development (development rate, duration of development), wing length, difference in left and right wing and mortality rate were investigated. Statistical tests were executed in R v4.3.3 (R core team, Foundation for Statistical Computing, Vienna, Austria). To compare the wing lengths, a linear mixed effects model (Gaussian) was used to distinguish the effects of exposure (EM and temperature) and diet, and account for the random effects by each of the replicates and the mosquito generations. When two-way interactions were included, the model was better according to the likelihood-ratio test ($\chi ^2 = 39.15, df = -3, Pr(> \chi ^2) = 1.61 \times 10^{-8}$). The effects on the absolute difference between left and right wings (asymmetry) for the different groups of exposure/diet/sex were assessed with a Kruskal-Wallis test.

The development time was determined for every individual insect and expressed in days for from L1 to pupation and from L1 to adult emergence. The log-rank test (Mantel-Cox test) was used to look pairwise at the difference in development time in a time-to-event analysis. First, the proportional hazards assumption was checked using the Schoenfeld residuals between the groups, see Supplementary Table S17. Combinations of the experimental conditions that are not shown in the Results, had non-proportional hazards (Schoenfeld p < 0.001).

From the development duration, the development rate could be calculated as 1/development duration. This development rate is expected to be uniform and linear with (water) temperature if all other conditions are kept constant^17^. The two control situations (HC and LC) only differed in temperature. The two EM exposed situations (LE and HE) differed in E-field strengths and as a consequence also in temperature of the water. If the RF-EMF exposure has only a temperature effect, the linear curves for the development rate over temperature for the control groups and for the EM exposed groups should be equal. A new distribution of 10000 slopes was calculated for the linear curves (LC-HC and LE-HE) for both diets, with two random points taken from the measured temperature (x-axis) and measured development rate (y-axis). These slope distributions were then compared by a pairwise t-test for unequal variance (Welch). A pairwise Wilcoxon rank sum test was also added to the Supplementary Information comparing development time between exposure conditions and diet.

Finally, the survival was shown in Kaplan-Meier curves with emerged adults censored. Mortality rate was calculated as number of deaths over the total of deaths and adult emergences for the 5 runs of the 4 experimental conditions and 2 feeding regimes. The mortality rates for the different groups were compared with a Kruskal-Wallis test.

## Results and discussion

Before the EM exposure experiments were conducted, a dedicated RC was built and characterized, ensuring controlled exposure. Next, numerical simulations of the RF-EMF exposure on the samples in the experimental conditions were made to obtain dosimetric information needed to understand at what dose the results of the exposure experiments occur.

During the experiment, 168 Petri dishes, each containing one larva, were exposed to 3.6 GHz (exposed groups) or were placed in the setup without the RF source on (control groups). In total, 4 experimental conditions were considered:

- Low Temperature Control (LC): 28.23 ± 0.07 $^\circ$C, RF off.
- Low EM Exposure (LE): 28.16 ± 0.07 $^\circ$C, 58.2 ± 1.0 V/m for the unloaded chamber, 46.2 ± 0.3 V/m for the loaded chamber.
- High Temperature Control (HC): 30.33 ± 0.01 $^\circ$C, RF off.
- High EM Exposure (HE): 29.95 ± 0.11 $^\circ$C, 278.9 ± 4.7 V/m for unloaded chamber, 182.6 ± 2.0 V/m for the loaded chamber.

The listed values indicate measured water temperature and the measured electric field strengths ± standard error, respectively. Note that high and low are relative terms. Due to dielectric heating by the RF-EMF exposure during the HE, an increase in water temperature occurred, while the ambient temperature settings were equal to the ones during LC. The temperature setting for HC was set to match the water temperature during HE.

### Reverberation chamber

The use of an RC to expose insects to RF-EMFs is a novel approach, it makes it possible to uniformly expose a large number of Petri dishes at the same time to EMFs. The constructed RC should operate at well-stirred conditions (i.e., exhibit uniform and isotropic EMFs)^24^ at 3.6 GHz. This was verified by studying the modified AD-statistic (Anderson-Darling) ($A^2_m$) for the stirred component of the measured power reflection coefficient $| S_{11}^{sti}|$ for a Rayleigh distribution and the first-order auto-correlation factor (*r*(1))^24^. The $A_m^2$ value should be below the threshold of 1.341 to be considered Rayleigh distributed over its N positions, and create a uniform field in the RC. The *r*(1) should be below the threshold depending on N to have uncorrelated stirrer positions. The two stirrers shown in Fig. 1a-b can rotate independently, three different configurations of the stirrer positions (N = stirrer 1 position $\times$ stirrer 2 position) were considered, 16$\times$16, 8$\times$8 and 8$\times$1, for which the $A_m^2$ and *r*(1) are shown in Fig. 1d in an unloaded chamber, i.e. with no samples in the working volume. For the 8$\times$8 case, the loaded condition was investigated too, where the working volume is loaded with 168 Petri dishes filled with water.

For 16$\times$16, the *r*(1) does not drop below the threshold at 3.6 GHz, making it unfit for operation. For all three unloaded conditions, the polynomial fit for $A_m^2$ is below the threshold at 3.6 GHz. For the 8$\times$8 and 8$\times$1 configurations, the $A_m^2$ suggests that $|S_{11}^{sti}|$ is Rayleigh distributed over the N measurements at 3.6 GHz, meaning a uniform EMF is established in the working volume^24,43^. However, when few stirring positions are considered (typically < 30), a Rayleigh distribution is not expected^35,51,52^ and the AD-test, even though it is a strong goodness-of-fit test, is not sufficient to validate the distribution in this case^40^.

Therefore, we added a third quantity $\sigma / \mu _{S_{11}}$, or coefficient of variation (*CV*), i.e the standard deviation of the measured $| S_{11}^{sti} |$ over all stirrer positions N, divided by the mean $| S_{11}^{sti} |$. When using the method of^24^, the same measurements can be used, promoting ease of measurements. For this distribution to be Rayleigh distributed, *CV* should be close to 0.523^40^. From the frequency of 3.6 GHz up to the highest measured frequency 5.9 GHz, the 95% interval of the *CV* is indicated with gray lines. A higher N has a *CV* closer to the ideal of 0.523, and has a smaller variance on *CV* (see 16$\times$16 vs. 8$\times$1), and thus is closer to an ideally Rayleigh distributed $|S_{11}^{sti}|$ over the N positions in the given frequency range. However, the advantage of higher N on the *CV* becomes small after N = 64 (Supplementary Fig. S7).

The value of *CV* alone cannot be used to validate whether the data is Rayleigh distributed, e.g. at the lower frequencies of the 16$\times$16 configuration. The combination of the three quantities presented in Fig. 1d ($A_m^2$, *r*(1) and *CV*) for the different N makes it possible to experimentally determine the lowest usable frequency for given N, and to choose a configuration of stirrer positions N. For the RC in this study using 3.6 GHz, the best configuration was 8$\times$8 according to these three quantities. (For more considered stirrer combinations N , see Supplementary Information Fig. S4-7.)

In^40^ and^53^, the standard deviation to mean ratio (or *CV*) as a function of frequency was also used to characterize the RC; however, not through the $S_{11}$, instead they used the *CV* for a Cartesian component of the electric field used in the standard method^35^, which ideally is also Rayleigh (or otherwise^40^) distributed. In these studies, the *CV* is higher than the Rayleigh characteristic value of 0.523 at low frequencies, while for the $| S_{11}^{sti}|$, the *CV* is around this value at all measured frequencies. The RC can be used in future experiments at different frequencies from the studied frequency range, given the $A_m^2$ and *r*(1) are below their respective thresholds, which is the case for 1.35-5.9 GHz for 8$\times$8.

For the exposure experiments, the 8$\times$8 configuration was the best option when all three graphs are considered for 3.6 GHz. The loaded chamber with 168 filled Petri dishes shown in Fig. 1d has very similar results as the loaded condition. Under these conditions, fewer reflections occur due to the interaction of the water with the RF-EMFs. Hence, field uniformity occurred also when the chamber was loaded^54,55^ with the 168 Petri dishes, outside of the Petri dish matrix. However, this does not imply that the individual Petri dishes also experienced a uniform field. Simulations are needed to further understand the exposure of the larvae in a system of multiple Petri dishes.

### Numerical simulations

Simulations using FDTD were conducted to determine the absorbed EM power ($P_{abs}$) by the larva in a Petri dish with water. For the dosimetry, it was chosen to work with whole-body $P_{abs}$^4^ rather than Specific Absorption Rate (*SAR*), since the mass and the density of the insect is not known, and the volume is often too small for calculating standardized localized *SAR* averages^47^.

#### One larva in Petri dish

First, a 3D model of the larva was created for the simulations. Different views of the new 3D model generated from $\upmu$CT-scans of a *Ae. aegypti* larva instar 4 are shown in Fig. 2a. Before $\upmu$CT-scans were conducted, the larva was critical-point-dried to prevent it from collapsing. Small damages were still found. However, a high level of detail was captured. During the larval stage, the larva molt from instar 1 to 4 (L1-4), growing in size. Therefore, the anatomical 3D model of L4 was rescaled to the lengths of L3, L2, and L1, representing the growth of instars (see Supplementary Table S1). The anatomical 3D models, as well as their analogous geometric spheroid models^5^, were placed in a 3D model of a Petri dish filled with water in different positions, see Fig. 2.a-d., representing the situation during the exposure experiments. The geometric vertical positions (least amount of simulations) was used as a basis for simulation uncertainties (Supplementary Information) and frequency dependency.

For the geometric models’ vertical positions of every instar and for the anatomical model of L4’s vertical position, the $P_{abs}$ as a function of frequency is divided by the volume of the respective 3D model for an incident root mean square (RMS) electric field strength of 1 V/m (Fig. 2.f. and Supplementary Fig. S8). The general trend shows a lower $P_{abs}$ for higher frequencies. This is different from insects in free space where the $P_{abs}$ increases with frequency, such as seen in^4,28^. However, in this case, the surrounding water will also absorb EM power and at higher frequencies, the penetration depth of the water is lower and EMFs of lower intensity will reach the aquatic larvae. At 3.6 and 6 GHz, the effect of the instar stage is small compared to higher frequencies. In future research on the *Ae. aegypti* larva under EMF exposure, other frequencies might be used and $P_{abs}$ can be estimated from the results given here if the frequency is in the studied frequency range. The exposure experiment in this study used a frequency of 3.6 GHz, at which the larvae of every instar experienced a similar dose per volume and it is not needed to determine the larval stage during the exposure experiment. Only a small difference is observed in the mean $P_{abs}$ of the anatomical model and the geometrical model of L4 in the vertical position in Fig. 2f (12% at 3.6 GHz and even less for the other instars). Hence, the geometric spheroid model is a good approximation for the anatomical 3D model based on $\upmu$CT-scans of an *Ae. aegypti* L4 larva, in line with^5^.

At 3.6 GHz, the EMF inside the Petri dish varies along the water and $P_{abs}$ becomes position-dependent. To investigate the effect of the larval position within the Petri dish on $P_{abs}$, two positions were considered in addition to the vertical position. The tilted position is a more realistic position during breathing, and the vertical position corresponds to the feeding behavior close to the bottom. The $P_{abs}$ in these situations are shown in Fig. 2g. The *P* *abs* can be rescaled for different incident RMS electric field strengths (see Supplementary Information), which was 46.2 V/m for this figure, corresponding to the field strength measured in the loaded RC during the experiment at conditions LE (instead of the unloaded RC, since the power absorption of the other Petri dishes is taken into account). The results are not normalized to volume and the L1 stage has a lower total $P_{abs}$ compared to later instars due to its smaller volume. In a breathing position near the surface (vertical and tilted position), the larva will experience more power absorption than at a feeding position where the larva is found near the bottom (horizontal position). During the experiment, the larvae moved freely in the Petri dish, and it is assumed that they all received a similar dose between the vertical and the horizontal position.

For the vertical position of the anatomical model, the cross sections of the electric field strength in the water and larva in the Petri dish are given in Fig. 2h for 8 incident plane waves (1 V/m). The wavelength of the EM field (8.3 cm) is of the same order of magnitude as the dimensions of the Petri dishes; hence, some resonance can be expected in the water^4^, which is visible in the cross sections. The difference between the point of highest and lowest mean electric field strength over the 12 plane wave simulations was a factor of 42 (Fig. 2i).

The numerical simulations involve uncertainties, detailed in the Supplementary Information. The primary sources are variations in the water level and the unmeasured dielectric properties. During the exposure experiments, the water levels could fluctuate to some degree, and the dielectric properties of the water could slightly be influenced by the addition of food for the larva. A limitation of this study is the lack of direct measurement of dielectric properties. However, the uncertainty analysis takes this into account, where It was seen that the position of the larva in the Petri dish and the instar of the larva had a larger impact on the whole-body $P_{abs}$ than other uncertainties studied.

For the experimental conditions HE, an electric field strength of 182.6 V/m (loaded RC) was applied. In this case, for the anatomical vertical position, a $P_{abs}$ of 1.04 $\upmu$W (or 16.5 $\upmu$W/mm$^3$) was calculated for L1 and of 0.13 mW (or 29.2 $\upmu$W/mm$^3$) for L4. For the experimental conditions LE, an electric field strength of 46.2 V/m (loaded RC) was applied. In this case, for the anatomical vertical position, a $P_{abs}$ of 66.7 nW (or 1.06 $\upmu$W/mm$^3$) was calculated for L1 and of 8.36 $\upmu$W (or 1.87 $\upmu$W/mm$^3$) for L4. When approximating the density of both water and larva with 1000 kg/m$^3$, a whole-body averaged *SAR* of 29.3 W/kg for L4 at HE conditions and 1.87 W/kg for L4 at LE conditions was found. For LE, this is lower than the ICNIRP basic restriction for local *SAR* for both head/torso and limb (averaged over 10 g, while the larva is actually lighter), but higher than the basic restriction for the whole-body averaged *SAR*. In HE conditions, the electric field strength is higher than the ICNIRP reference level and the *SAR* is higher than the whole-body and local basic restrictions for humans^47^.

#### Petri dish matrix

During the experiments, 168 Petri dishes were placed in the RC simultaneously in a matrix of 7 rows, 6 columns and 4 levels. The effect of Petri dish placement was considered with extra simulations on all 168 Petri dishes. Since the incidence angles of the original 12 plane waves result in high shadowing effects (see Fig. 2h), extra simulations were considered for a total of 52 incident plane waves, for Petri dishes only filled with water. The Petri dishes with the highest $P_{abs}$ by the water were found to be the ones on the corners (see Fig. 2i). The values of the highest and lowest $P_{abs}$ are 1.85 $\upmu$W and 0.22 $\upmu$W, respectively for an incident electric field strength of 1 V/m. Because of shadowing, the 12 plane waves used in the simulations on a single Petri dish are thus not accurate for all Petri dishes, but can serve as a balance for all Petri dishes. The measurement of electric field strengths in the loaded RC happened at the side and previous $P_{abs}$ values based on these measurements are thus more reliable for the Petri dishes on the sides and serve as an upper limit for the Petri dishes in the middle.

Due to $P_{abs}$ by the water, dielectric heating of the water can occur. In^25^, EMF induced dielectric heating of Petri dishes depending on the size of the sample and the position. Here, we did not only consider the size and position of the larva within the Petri dish, but also the position of the Petri dish in the exposed system^54^. Some shadowing effects are expected for Petri dishes placed close to each other and they can experience different $P_{abs}$ as shown in the simulations. To compensate for shadowing, the larvae can be rotated throughout the experiment, as was done for the levels of the Petri dish matrix.

### Exposure experiments

**Fig. 3:**
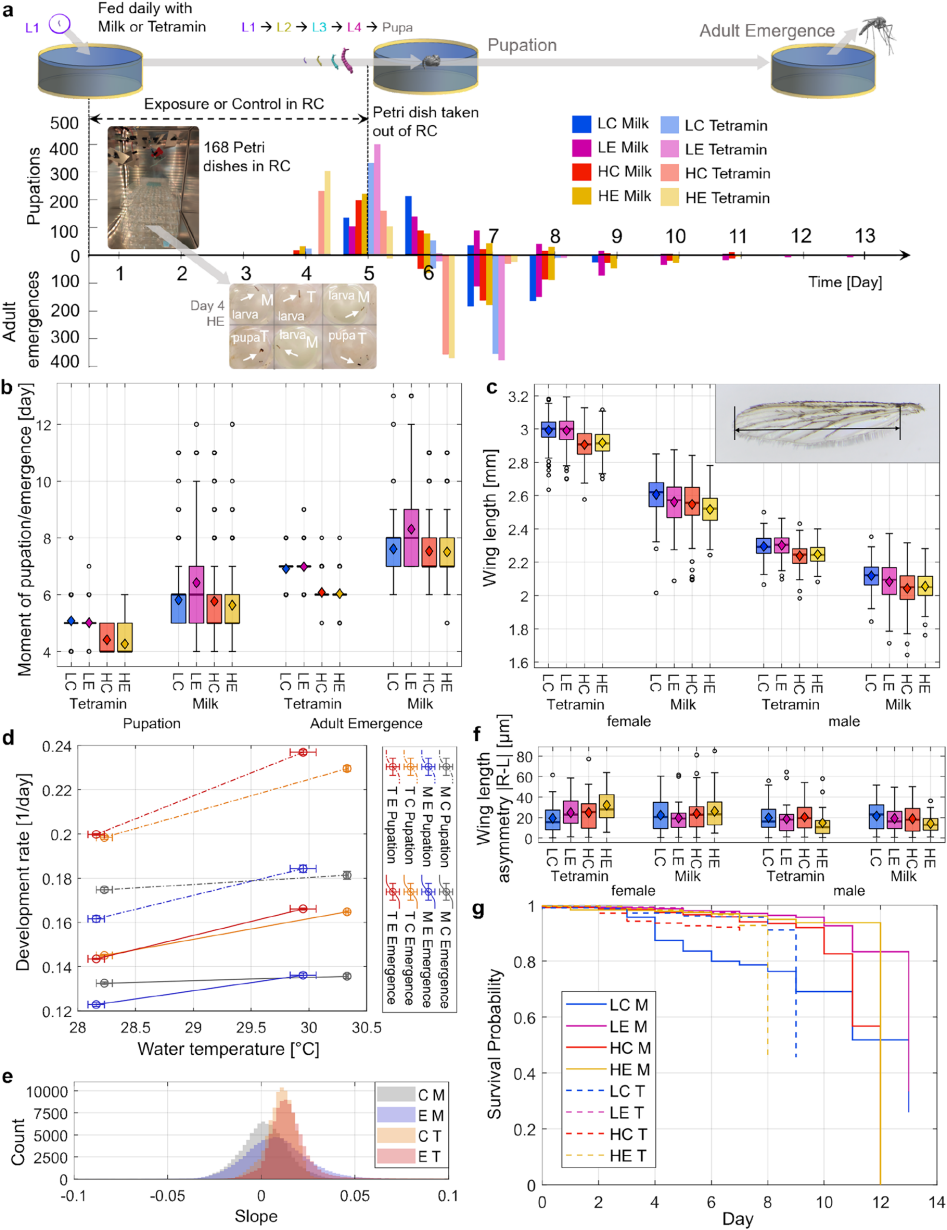
The protocol and effects of the electromagnetic exposure on *Aedes aegypti*. (**a**) Schematic overview of experiment with the number of pupations and adult emergence combined for all 5 replicates as a function of time per feeding regime and experimental conditions: Low-temperature control (LC), Low EM Exposure (LE), High-temperature control (HC), High EM Exposure (HE). (**b**) Moments of pupation and moments of adult emergence, for the different experiments and feeding regimes. Per experiment and feeding regime, 840 insects were used. (Box: 25th to 75th percentile, Thick horizontal line: median, Circles: outliers, Diamond: mean.) (**c**) Wing lengths measured across the different experiments, the two feeding regimes and sexes are also separated. In the top right, the measurement of the wing length is shown. (**d**) Development rate for the different experiments as a function of temperature. The error bars indicate the standard error. (E: exposure (LE and HE), C: control (LC and HC), M: Milk, T: Tetramin). (**e**) Counts of slopes calculated between 100k random two points taken from temperature and random two points of development rate of pupation (one LC/LE and one HC/HE). The moment of pupation was uniformly distributed between days to make the distributions less discrete for visual reasons only. (**f**) Asymmetry given by the absolute difference between the left and right wing length. (**g**) Kaplan-Meier survival curves, data censored for adult emergence.

The RF-EMF exposure experiments were executed after characterizing the RC and determining the dose. For every experimental condition, 168 Petri dishes with one *Ae. eagypti* L1 larva each, were placed inside the RC on day 0 and removed on day 5. Every day, half of the larvae were fed with milk powder (M) and the other half with Tetramin fish food (T). Every experimental condition was repeated 5 times. The entire flow of the experiment is shown in Fig. 3a alongside the frequency of pupation and adult emergence per day for both diets and the four experimental conditions (LC, LE, HC and HE). The researchers were not blinded to the experimental conditions. However, wing length, moment of emergence, and mortality cannot be subjectively changed by the observer.

An increase in temperature was measured for HE due to dielectric heating caused by the EM exposure^20^. The EM power absorption normalized to the volume is higher for the larva than for the water at 3.6 GHz, this is indicated by the E-field strength in the larva from the simulations shown in Fig. 2h (and quantified in the Supplementary Information, see Fig. S9). The dielectric heating will increase the temperature of the larva slightly more than that of the water, heat will dissipate to the water until an equilibrium is reached. At HE, L4 larvae absorb 0.11 mW in vertical position, which is considerable. For LE, a temperature rise was not detectable.

#### Development time

The day of pupation and adult emergence for each mosquito was recorded (Fig. 3.b. and Supplementary Fig. S12 and Table S8). It is clear that a milk diet and a lower temperature, resulted in a slower development. In Table 1, the time to event (pupation/adult emergence) was compared pairwise. The proportional hazards assumption is not met for Tetramin-fed HE vs. HC pupation (Supplementary Information, see Table S17) and this result is thus not reliable. For all pupations, there was a difference in development timing (Log-rank p < 0.05). For milk-fed LC vs. LE, the difference in time to adult emergence was the most statistically significant and the difference in timing can be seen in Fig. 3.b. Also, the stronger Tetramin-fed larvae of LC and LE show a significantly different development timing, again the LE being slower than LC. For adult emergence of larvae fed with milk, there was no significant difference for HC vs. HE. Similar conclusions were found for a pairwise Wilcoxon rank sum test, see Table S16.

**Table 1:** Log-rank test for pupation or adult emergence for different experimental conditions and diet.

Event	Diet	Exp. cond.		$\chi ^2$	df	p-value
Pupation	M	LC	LE	55.7	1	< 0.001
Pupation	M	HC	HE	6.4	1	0.010
Adult em.	M	LC	LE	66.5	1	< 0.001
Adult em.	M	HC	HE	0.3	1	0.600
Pupation	T	LC	LE	6.1	1	0.010
Adult em.	T	LC	LE	8.0	1	0.005
Adult em.	T	HC	HE	2.3	1	0.100

Abbreviations: Experimental conditions (Exp. cond.), Milk (M), Tetramin (T), Low temperature control (LC), High temperature control (HC), Low exposure (LE), High exposure (HE), degrees of freedom (df).

Another way to look at the development time is by taking the rate of development (1/days) and plotting it against temperature, see Fig. 3.d. To check whether the slopes of the RF-EMF exposed (LE and HE) and the control (LC and HC) lines are different, distributions were simulated by calculating 100k slopes per line by taking a randomly measured water temperature in the LE vs. LC and the HE vs. HC condition, and a randomly measured developmental rate in the LE vs. LC and HE vs. HC condition, shown in Fig. 3e. The four slope distributions for adult emergence or pupation were then compared using an unpaired t-test for unequal variance. The slopes of the control groups are significantly different from those of EM exposure, except for the pupation when fed with Tetramin (t-value = 0.17, df = 1.89E+05, p-value = 0.86). The difference in slope of development rate as a function of temperature can be either caused by non-thermal effects of the EM exposure, or by a higher temperature of the larva than the water due to dielectric heating.

#### Wing length

Wing length of the adults right after emerging were measured as a proxy for body size and shown in Fig. 3c. (and Supplementary Table S9-11). Female wing lengths were modeled with a Linear Mixed-effects Model (LMM), the results are given in Table 2. An LMM for the wing lengths of the male adults was included in Supplementary Information Table S13 along with the LMM for female wing lengths with other reference baselines (intercept) Table S12.

**Table 2:** The results for linear mixed-effects model (Gaussian) for wing length for female *Ae. aegypti*.

Term	Estimate	Std.Error	t-value	df	p-value	Sign.
(Intercept)	2992.23	21.08	141.93	16.89	2.01E-27	***
type HC	-82.14	28.42	-2.89	17.20	1.01E-02	*
type HE	-75.63	28.28	-2.67	16.88	1.61E-02	*
type LE	2.18	28.18	0.08	16.62	9.39E-01	
Food M	-386.16	12.14	-31.82	1402.27	1.02E-167	***
type HC:Food M	15.41	16.67	0.92	1405.68	3.56E-01	
type HE:Food M	-13.49	15.95	-0.85	1401.45	3.98E-01	
type LE:Food M	-45.02	15.59	-2.89	1401.87	3.94E-03	**
Random par: Run ID		sd = 38.86	(intercept)			
Random par: residuals		sd = 101.22	(Observation)			
Significance codes: < 0.001 ‘***’ 0.001 ‘**’ 0.01 ‘*’ 0.05 ‘.’ 0.1 ‘ ’ 1.						
Baseline (intercept): LC, Tetramin, female.						

Abbreviations: Milk (M), Tetramin (T), Low temperature control (LC), High temperature control (HC), Low exposure (LE), High exposure (HE), standard error (Std.Error), degrees of freedom (df), significance (Sign.), standard deviation (sd).

Adults emerging from Tetramin-fed larvae were larger by 13% than those reared on milk. The HE and HC experiments led to a shorter wing length compared to LC, with estimated reductions of approximately 76 $\upmu$m and 82 $\upmu$m, respectively, with a (p = 0.01). while LE is not significantly different from LC. The interaction of diet with RF-EMF exposure means a larger influence on the wing length in the LE group when fed with milk.

A possible measure of environmental stress during development is asymmetry in wing length in insects from the two sides of the mosquito^56^. In^22^, the rearing temperature affected the wing shape and fluctuating asymmetry in *Culex pipiens*. In our study, no influence of temperature, diet and RF-EMF exposure on the difference between left and right wing length was observed (Kruskal-Wallis test: $\chi ^2$ = 3.19, df = 7, p = 0.87), see Fig. 3f, likely because the difference in temperature was not as drastic as in^56^.

#### Mortality

The Kaplan-Meier curves for the different experimental conditions are shown in Fig. 3g demonstrating similar trends, except for the LC milk condition. The mortality rate is defined as the number of deaths over the total number of adult plus deaths. The overall mortality rate was 6.0% (see Supplementary Information for the mortality rate per experimental condition).

In^17^, an effect on survival was found by the diet; however, here no effect on mortality was detected between all diets and experimental conditions (Kruskal-Wallis: $\chi ^2$ = 10.80, df = 7, p-value = 0.15). This might be explained by the absence of competition for food, since larvae were individually reared. Also, for both HE and LE, no effect on mortality rate was found.

#### Discussion

From the EM exposure experiments, effects were found for diet, temperature, LE and HE exposure. The effect of diet is clear from Fig. 3 and Table 2 on both wing length and development timing. Nutritional stress caused by the low concentrations of skimmed milk will result in smaller mosquitoes and in slower development relative to the optimal diet of Tetramin^17,21,57^. A mosquito larva needs a minimum amount of nutrition to trigger hormonal development cascades to evolve to the next life stage^17,58–60^.

A faster development was recorded, and smaller wing lengths were measured for the mosquitoes of the experiments with relatively higher water temperature ($\sim 30^{\circ }$C), HC and HE, compared to experiments with lower temperatures ($\sim 28^{\circ }$C), LC and LE. The critical larval weight required by the larva for metamorphosis is temperature-dependent and suboptimal temperature affects the proportion of body weight that is attributed for storage of nutrients^60^. Lower nutritional reserves can shorten the life of a mosquito and smaller females produce fewer eggs, but can also travel further and bite more frequently; hence, lead to a higher vectorial capacity^19,60–62^. The relation of temperature, diet and its interaction to larval development has been shown previously^17,63–65^.

Significant effects of the HE were recorded for the development (duration and size) compared to LC, which was also true for HC, while HC and HE showed similar results relative to each other. This suggests that the dielectric heating of the larva caused by the EMFs has a similar effect as applying external heating and the main effects on development of HE is presumed to be thermal due to dielectric heating. Note that at HE relatively high electric fields were generated in comparison to the ICNIRP reference levels at 3.6 GHz, and the simulated absorbed EM power of 0.11 mW by the larva (L4 vertical) is considerable. In field conditions, significant temperature variations can affect larval development^1,17,66^. When high EMFs occur, dielectric heating can further elevate temperatures beyond the natural variation. However, it is unlikely that *Ae. aegypti* larvae will encounter EMFs of the same electric field strength as in HE conditions at 3.6 GHz.

The LE experiments had a lower field strength, below the ICNIRP 30 minute average general public reference level^47^, and a simulated absorbed EM power of 66.7 nW and 7.2 $\upmu$W for the vertical orientated L1 and L4 larva, respectively. Under this exposure, some delay was found in development compared to LC, especially for the weakened milk-fed groups, which is unlikely to be a thermal effect. The first developments of LC and LE happened on the same day, however, for the milk-fed larvae, the spread of the transition timing was larger in the LE groups (See Supplementary Fig. S12). and some caution is needed for batch effects in these less stable milk-fed groups. The effect on development duration at HE, which is believed to be predominantly thermal, is opposite in direction and stronger in magnitude than the effect found at lower RF-EMF exposure for milk-fed larvae. A significant difference in wing length was not found for LE compared to LC in the linear mixed effects model.

In^15^, *Ae. aegypti* eggs exposed to 900 MHz and 18 GHz also had a slightly slower development duration from egg to adult. However, the exposure levels in^15^ are not indicated; hence, it is impossible to compare with these results. In^16^, the early life stage of *Ae. aegypti* were exposed to 900 MHz and 18 GHz, showing a faster larval development at 18 GHz for 30$^\circ$C and a slower larval development at 18 GHz for 35$^\circ$C. No effect was found on larval development for 900 MHz. However, exposure level and dose were not reported, making it impossible to interpret and compare these results. In^67^, a delayed development was found after exposing *Drosophila melanogaster* larvae to 10 GHz at electric field strengths ($\tilde{3}{.4}$ V/m) lower than LE. In^9^, on the other hand, a shorter development time was recorded for long term exposure of *D. melanogaster* at 3.5 GHz at fields comparable to or lower than LE. However, since the fruit flies were not aquatic but comparable in size to the *Ae. aegypti* larva L2, they might have encountered a higher $P_{abs}$ for a similar field strength than the mosquito larvae at LE. The authors concluded that RF environmental stress has an impact on thermal stress and ecdysteroid signaling pathways promoting the development of the fruit fly, among other effetcs^9^. The delayed or faster development of the fruit flies in^9,67^ seem dose-dependent and appear to be in line with our results. In^11^, honey bee queens exposed to 900 MHz showed higher mortality during the pupal development and *Tenebrio molitor* larvae exposed to high electromagnetic fields generated between electrodes at 39 MHz, presented morphological changes after development in^68^. For the mosquitoes however, we did not find any effect of RF-EMF exposure on mortality, asymmetry or malformations.

Up to now, no thermal thresholds have been established for RF-EMF exposure of invertebrates. Our results show that RF-EMF absorption in an insect, when averaged over the 4 instars and the 2 main positions, i.e. $1.9 \times 10^{-5}$ W, corresponding to HE, induces dielectric heating and developmental effects. Additionally, we showed that an absorbed power of $1.2 \times 10^{-6}$ W corresponding to LE, induces developmental effects, especially for weakened insects. We think that it is unlikely that such absorptions will occur in aquatic conditions in nature. However, it seems probable that these absorptions can occur in aerial or terrestrial insects. The authors of^28^ show that adult mosquitoes absorb $4 \times 10^{-9}$ W for an exposure of 1 V/m at 60 GHz. Hence an exposure of 17 V/m (below INCIRP reference level) would induce $\sim 1.2 \times 10^{-6}$ W in this insect, and 69 V/m would induce $\sim 1.9 \times 10^{-5}$ W. For other insects in the far field, an RF-EMF absorption of $\sim 1.9 \times 10^{-5}~W$ needs a lower incident electric field strength^4,5,27,28^, e.g. 6.9 V/m for a black field cricket exposed at 6 GHz^5^. For the LE condition of $\sim 1.2 \times 10^{-6}$ W, the black field cricket only needs 1.7 V/m. However, in order to experience the same average *Pabs/V* as experienced by the mosquito larvae during the LE conditions (0.75 $\upmu$W/mm$^3$ for an incident field strength of 46.2 V/m), the larger cricket would need to experience a higher incident field strength of 45.1 V/m. In the near field of the antenna, where flying insects can appear, the absorption could be higher^69^. For example, at 5 cm and 6 GHz, a western honey bee experiences an RF-EMF absorption of $\sim 2 \times 10^{-4}$ W for an accepted power by a dipole antenna of 1 W.

## Conclusion

A large study on the development of *Aedes aegypti* mosquito larvae under 3.6 GHz radio frequency electromagnetic field (RF-EMF) exposure was established. As exposure setup, a reverberation chamber was built and characterized, demonstrating the well-stirred condition. In the setup loaded with water, a uniform field was created with an electric field strength of 46.2 V/m and 182.6 V/m.

Using numerical simulations, the absorbed dose for the larvae was calculated for the two exposure levels in different positions for a geometric and an anatomical 3D model. The latter was created using micro computer tomography scans of a real larva. We demonstrated that a feeding larva near the bottom is expected to experience less electromagnetic power absorption than a breathing larva near the surface. The breathing positions resulted in a power absorption of 6.67 $\times 10^{-8}$ W for larval instar 1, and of 7.19 $\times 10^{-6}$ W for instar 4 at 46.2 V/m. For 182.6 V/m, this resulted in a power absorption of 1.04 $\times 10^{-6}$ W to for instar 1 and of 1.12 $\times 10^{-4}$ W for instar 4.

The two exposure levels resulted in different effects on the insect. For the lower exposure level, no difference in wing length was found. However, a slower development occurred after exposure for 5 days of larvae fed with diluted milk to weaken them. The stronger larvae fed with Tetramin seemed not affected by the low exposure and the nutritional diet led to a faster development. At the higher exposure level, dielectric heating was observed and wing length measurements indicated smaller adults as a result of the thermal effect. The effect on development duration at this exposure level was stronger than the effect on development duration that was observed at lower exposure levels and an accelerated development was detected. Mortality and wing length asymmetry were unaffected by RF-EMF exposure.

We consider it highly unlikely that aquatic immature invertebrates do encounter these RF-EMF levels that might induce developmental effects in nature. On the other hand, our study does demonstrate that it is possible to induce delayed development and dielectric heating causing thermal effects with power absorptions that can occur in terrestrial and aerial insects. To the best of our knowledge, this is the most extensive study of RF-EMF effects on insect development including dosimetry, exposure setup development and calibration, experimental biology, and statistical analysis. These results are very important because they can establish a basis for thresholds and a dose-response relationship for developmental effects of RF-EMF exposure in insects.

## Electronic supplementary material

Below is the link to the electronic supplementary material.

Supplementary Material 1

## Data Availability

The datasets used and/or analysed during the current study available from the corresponding author on reasonable request. The 3D model generated and/or analysed during the current study is available in the Zenodo repository, https://doi.org/10.5281/zenodo.13881907.
