# Supplementary material for: Exposure to 5G-NR electromagnetic fields affects larval development of Aedes aegypti mosquito
Source: Sci Rep. 2025 Dec 25;16:2972. doi: 10.1038/s41598-025-32816-y (PMC12828023; doi:10.1038/s41598-025-32816-y)
Supplement: Supplementary file 1 — Supplementary Material 1 [file 41598_2025_32816_MOESM1_ESM.pdf]

Supplementary Information

Exposure to 5G-NR electromagnetic fields affects larval development of Aedes aegypti mosquito

Eline De Borre, et al.

Corresponding authors: Eline De Borre, eline.deborre@ugent.be;
Arno Thielens, athielens@gc.cuny.edu.

This file includes:

Fig S1-S12
Table S1-S16
Supplementary text:

1. **Antenna used in reverberation chamber**
2. **Modes and resonance mode frequency RC**
3. **Q-factor RC**
4. **Additional Q-factor calculations, based on BWQ**
5. **Different configurations of stirrer positions**
6. **Coefficient of variance as a function of N**
7. **K-factor of reverberation chamber**
8. **Dimensions of 3D larva models**
9. **Dielectric properties**
10. **Rescaling of absorbed power**
11. **Electric field at different frequencies**
12. **Absorbed power by the water**
13. **Simulation uncertainties**
14. **Preliminary experiments**
    a. **Container**
    b. **Diet**
15. **Day of event per experiment run**
16. **Wing length per experiment run**
17. **LMM wing length female and male**
18. **Difference between runs**
19. **Mortality rate**
20. **Wing length Asymmetry**
21. **Pairwise Wilcoxon rank sum test on development time**
22. **Proportional hazard assumption**
23. **Sex ratio**

1. Antenna used in reverberation chamber

A half wavelength dipole antenna of total length of 32.1 mm is used during the exposure of larvae. The measured power reflection coefficient $|S_{11}|^2$ of this dipole is shown in Fig. S1 for one position of the stirrers in the unloaded Reverberation Chamber (RC). The $|S_{11}|^2$ for the dipole antenna in a loaded RC with 168 Petri dishes filled with water, is also given in Fig. S1. It is clear from the comparison of the two graphs, that the petri dishes absorb RF-EMFs as the interference patterns reduce in magnitude. The $|S_{11}|^2$ of the broadband antenna in the RC, used during characterization of the RC, is also given for one position of the stirrers.

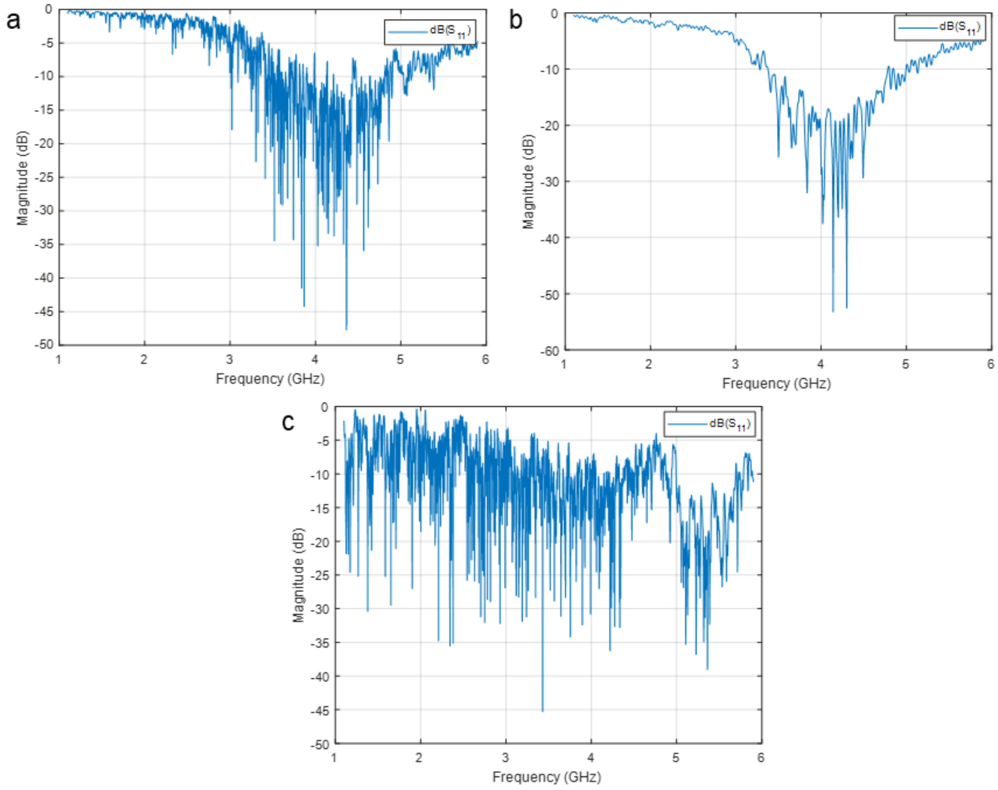

Fig. S1. S_{11} of (**a**) dipole antenna in unloaded RC, and (**b**) in the loaded RC, and (**c**) of the broadband antenna in unloaded RC.

2. Modes and resonance mode frequency RC

To estimate the size needed for an overmoded RC at the desired frequency, the resonance mode frequencies were determined using:

$$f_{mnp} = \frac{c}{2}\sqrt{\left(\frac{m}{L}\right)^2 + \left(\frac{n}{W}\right)^2 + \left(\frac{p}{H}\right)^2} \qquad (S1)$$

With *m*, *n* and *p* integers, *L,W* and *H* the dimensions of the RC, f_{mnp} the cavity resonance mode frequency and c the speed of light. The fundamental modal frequency (f_{011}) can be calculated from *L* = 43.6 cm, *W* = 46.6 cm and *H* = 60 cm, giving 407.3 MHz. A lowest usable frequency (*LUF*) can be estimated as 3 × f_{011}. A next step is to determine the number of modes (*N(f)*) in the cavity with these dimensions at the operating frequency, using:

$$N(f) \approx \frac{8\pi}{3} \times L \times W \times H \times \left(\frac{f}{c}\right)^3 - (L - W + H) \times \frac{f}{c} + \frac{1}{2} \qquad (S2)$$

With *N(f)* the number of modes for a certain frequency *f*. An RC is considered overmoded if N > 100. The lowest frequency corresponding to *N* = 100 for *L* = 43.6 cm, *W* = 46.6 cm and *H* = 60 cm is 1.1411 GHz. At 3.6 GHz the number of modes reaches 1751.
All previous equations in this section can be found in (Yousaf, *et al*. 2020).

3. Q-factor of RC

An interesting quantity in the characterization of the RC is the quality factor (Q-factor), which can be used to determine the degree of modal overlap, decay time of a signal and field strength inside the RC (Arnaut. 2003). The composite or effective Q-factor can be calculated using the S_{11} parameter using the following equation (Besnier, *et al*. 2015):

$$Q = \langle |S_{11} - \langle S_{11} \rangle|^2 \rangle \frac{Z_0 \omega \varepsilon V}{(\lambda^2/4\pi)(1 - |\langle S_{11} \rangle|^2)^2 \eta^2} \qquad (S3)$$

With Z_0 the free space impedance, ω the angular frequency, ϵ the permittivity, *V* the volume of the RC, λ the wavelength and η the antenna efficiency. The antenna losses by the antenna are neglected and the η can be approximated by 1. The average of the S_{11} parameter is over the N stirrer positions. The results are shown in Fig. S2 for N = 256 in the unloaded case when the broadband antenna was used.

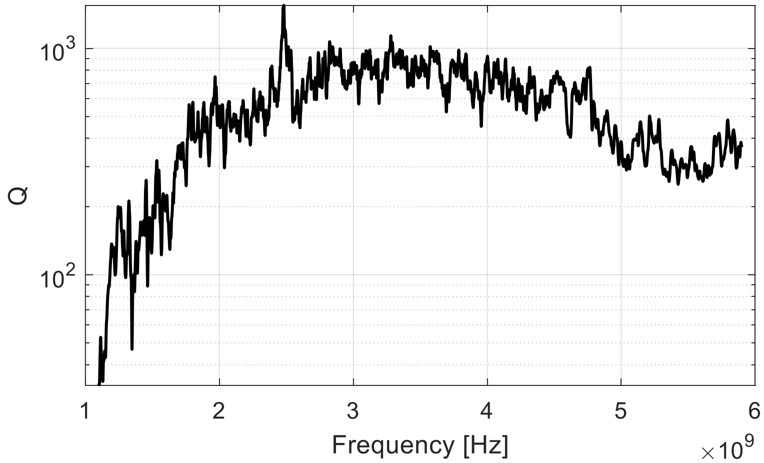

Fig. S2. Q-factor for the unoaded RC.

Q must be higher than a threshold Q_{thr} for an effective RC (Holloway, *et al*. 2015).

$$Q_{thr} = \left(\frac{4\pi}{3}\right)^{2/3} \frac{V^{1/3}}{2\lambda} \tag{S4}$$

Which is 7.74 for this RC.

4. Additional Q-factor calculations, based on BW_Q

From the composite Q-factor in the unloaded case, the modes (M) in inside the Q-factor band width $BW_Q = f_{l,m,n}/Q$ were determined using (IEC. 2011; Krauthauser, *et al*. 2012):

$$M = \frac{8\pi V f^3}{c^3 Q} \tag{S5}$$

Making it possible to determine the required number of independent samples for a field uncertainty of 6 dB at a confidence level of 95% (IEC. 2011; Krauthauser, *et al*. 2012). At 3.6 GHz, the number of stirrer positions required is 22 as can be seen in Fig. S3. V is the volume of the chamber, f is the frequency and c the speed of light.

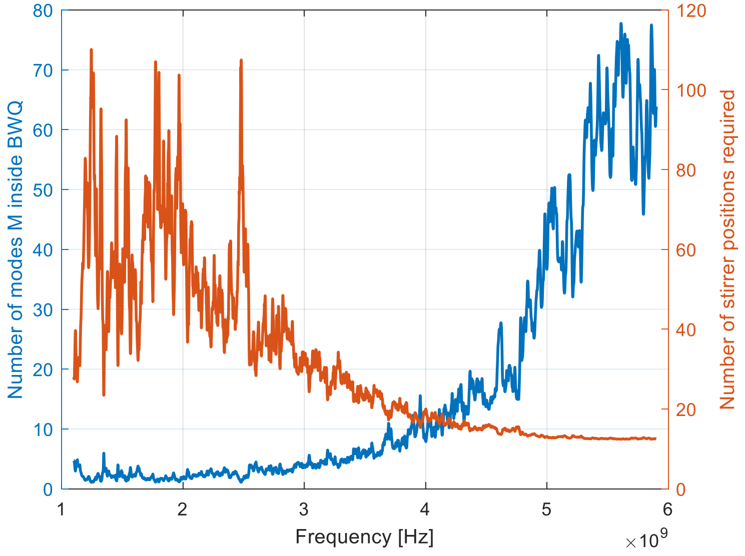

Fig. S3. Calculated number of stirrer positions required for a field uncertainty of 6 dB, based on the number of modes inside BW_Q.

5. Different configurations of stirrer positions

The RC contains two different stirrers, each can rotate stepwise 360° in N steps independently. Note that for all stirrer positions N, different configurations exist, e.g. 8×1 (versus 1×8) has one stirrer not moving. In such a scenario, it can be chosen which stirrer is fixed, and the position of this fixed stirrer can be altered. The different possible configurations all led to similar result, but the lowest usable frequency (*LUF*) can alter

slightly. Examples for the 8×8 configuration with different starting angles of one of the stirrers are shown below can be found in Fig. S4.

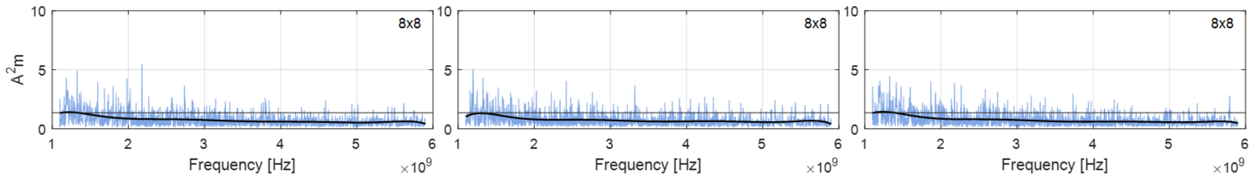

Fig. S4. The modified AD-statistic (A_m^2) for 8×8 stirrer positions with different starting points. S_{11} measurements with broadband antenna.

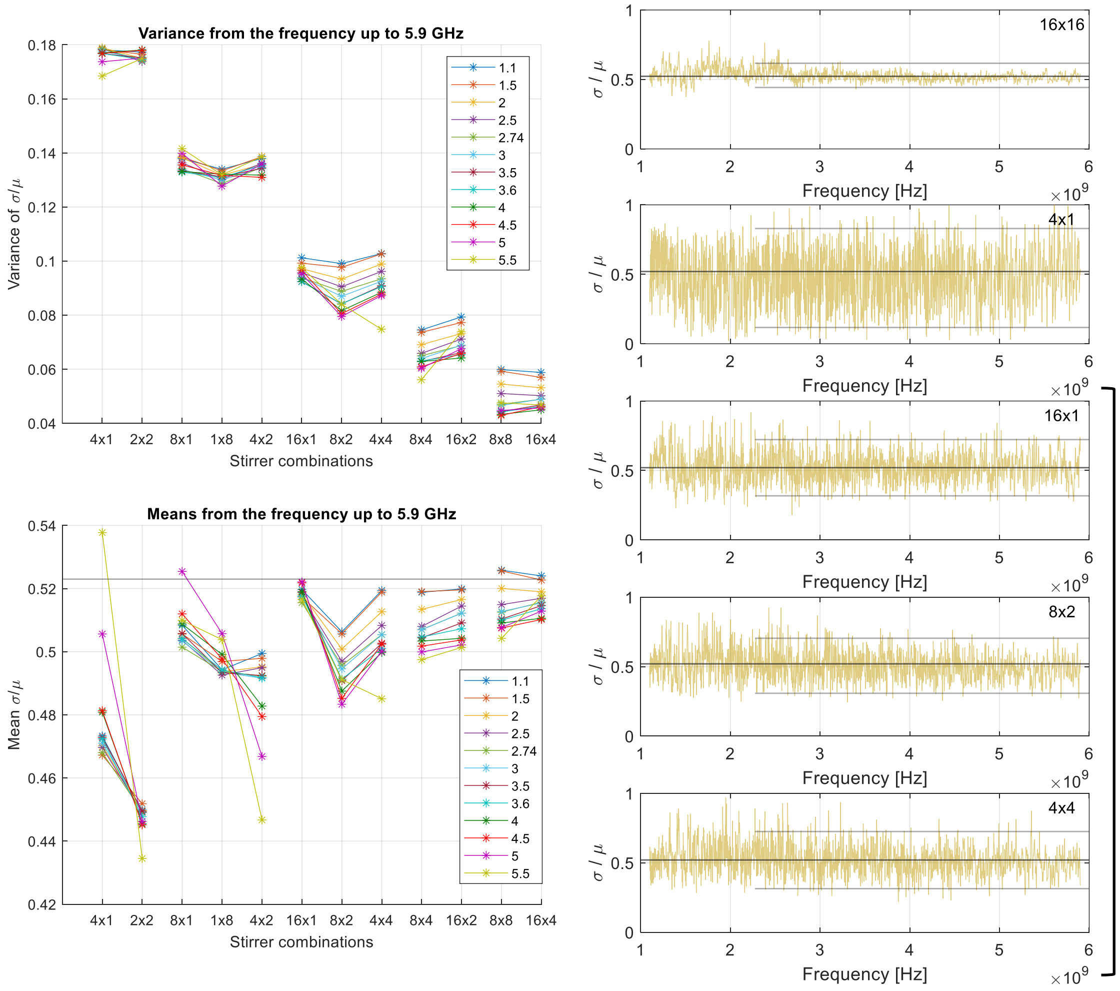

Fig. S5. On the left, the Variance and mean of $\sigma/\mu_{S_{11}}$ of the $|S_{11}|$ at each frequency over a range from the frequency indicated in the legend in [GHz] up to 5.9 GHz. On the right, the $\sigma/\mu_{S_{11}}$ of different stirrer combinations, in the bottom 3 plots are all combinations for N=16. S_{11} measurements with dipole antenna.

For the stirrer configurations used in the paper, the *LUF* can be determined. The A_m^2 polynomial fits of the measurements intercepts the threshold at 3 GHz for 16×16 and stays below the threshold for 8×1 and 8×8. For 16×16, the *r(1)* (shown in the paper) does not drop below the threshold at 3.6 GHz, hence, it is not suited for operation at this frequency. For the two other configurations, the *r(1)* is below the threshold for all measured frequencies. Looking at both A_m^2 and *r(1)*, the *LUF* is < 1.1 GHz for 8×1, 1.36 GHz for 8×8, and > 5.9 GHz for 16×16. However, the AD-statistic is not reliable for a small number of stirrer positions like 8×1 (Lemoine, *et al*. 2007).

For all possible 8×8, the highest value of *LUF* was found to be 1.36 GHz for the different measurements (see Fig. S4). We chose to work with this frequency as *LUF*, so the *LUF* was acceptable for all configurations of 8×8. A few examples of equal N but different amount of positions per stirrer are given below: 4×4, 16×1 and 8×2, all with a total N = 16.

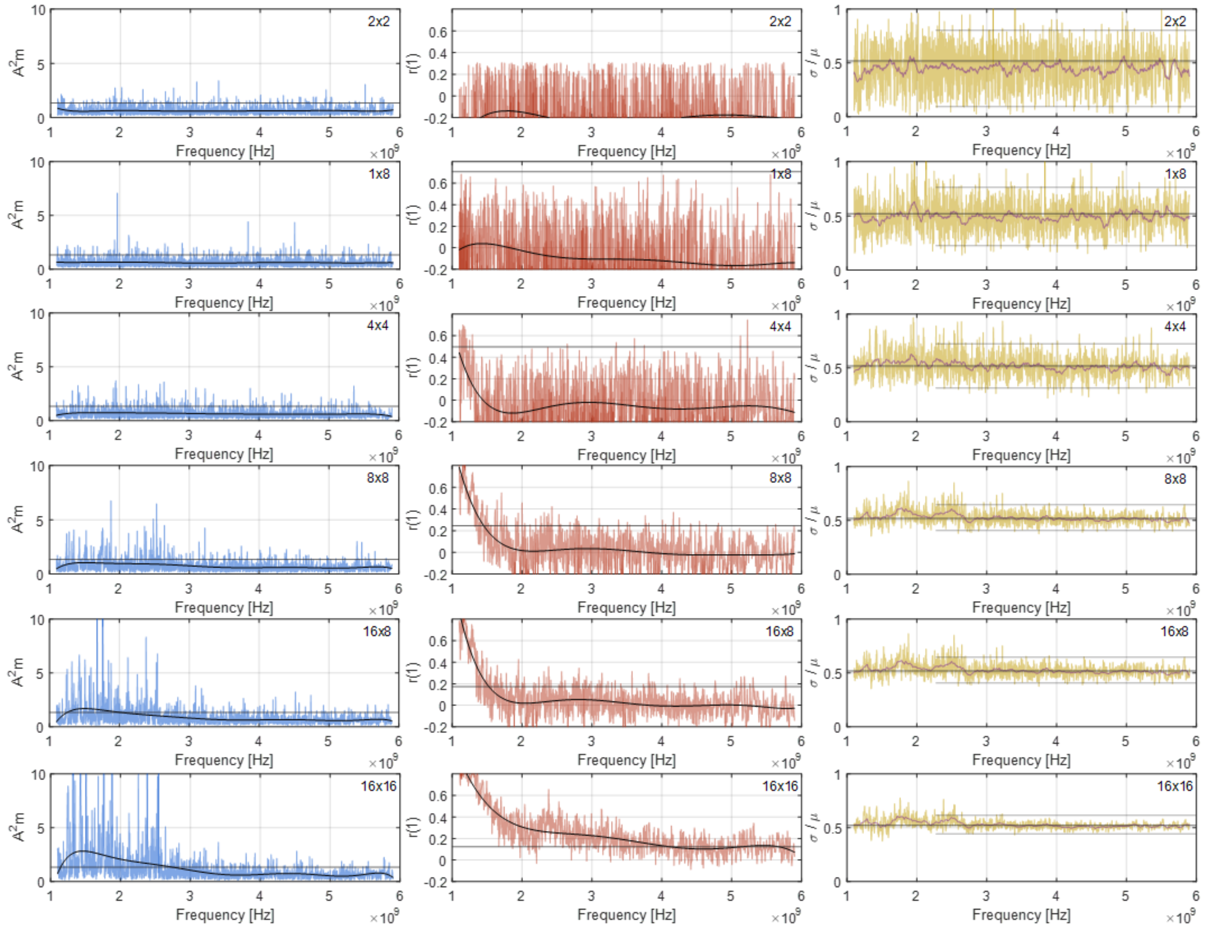

Fig. S6. A_m^2, *r(1)* and *CV* of different stirrer configurations. *S₁₁* measurements with dipole antenna.

In Fig. S5, It can be seen that the difference in variance of $\sigma/\mu_{S_{11}}$ (or *CV*) is small for a fixed N. However, the mean is more influenced by the different configurations of stirrer positions of a fixed N, particularly for small N. For start frequencies (see legend) close to the stop frequency (5.9 GHz) of the interval, fewer points are available and the result becomes unstable. Note that 8×1 keeps one stirrer fixed, while 1×8 keeps the other one fixed.

In Fig. S6, the A_m^2, *r(1)* and *CV* graphs are shown for some more values of N and measured with the dipole antenna. Note that the results are similar to those measured with the broadband antenna.

6. Coefficient of variance as a function of N

In Fig. S7, the Variance of the coefficient of variance (*CV*) of the $|S_{11}^{sti}|$ is given for different amount of stirrer positions N = 4×1, 8×1, 8×2, 8×4, 8×8, 16×8 and 16×16 in the unloaded RC. The frequencies shown in the legend are the starting frequencies for the range of frequencies over which the variance is taken, with 5.9 GHz the highest frequency of the range. For different starting frequencies over which the variance is taken, the variance is similar.

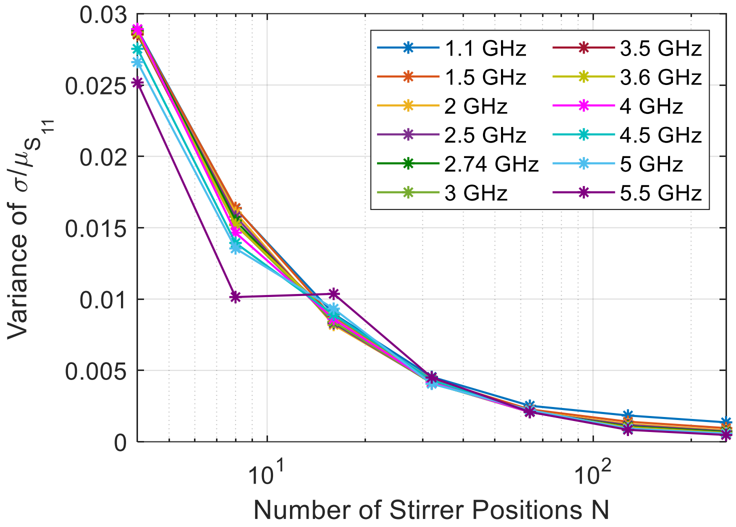

Fig. S7. Variance of the *CV* of the $|S_{11}^{sti}|$ from the frequency in the legend up to 5.9 Gz.

From Fig. S7., it is clear that a higher N has a *CV* closer to the ideal of 0.523, as expected, and has a smaller variance on *CV*. It is thus closer to an ideally Rayleigh distributed $|S_{11}^{sti}|$ over the N positions in the given frequency range. At N = 16, the variance is below 0.01 and at N = 32, the variance drops below 0.005. For N higher than 64, the difference in variance of *CV* becomes small, particularly between N = 64 and 128.The benefit of using an N higher than 64 is considered minimal in this exposure setup.

7. K-factor of the reverberation chamber

The Rician K-factor determines the ratio of unstirred to stirred power in the reverberation chamber and can be estimated from the S_{21} parameter (Chen, *et al.* 2011; Senic, *et al*. 2016), using:

$$K = \frac{|\langle S_{21} \rangle_N|^2}{\langle |S_{21} - \langle S_{21} \rangle|^2 \rangle_N} \qquad (S6)$$

If the EMF in the RC was perfectly stirred, hence Rayleigh distributed, the K-factor would be 0. For different numbers of stirrer positions, different K-factors were found. For 16×16, 8×8, and 8×1, K-factors of 0.14, 0.65, and 4.21, were found, respectively. Similar to what was shown for the coefficient of variation, a higher N will result in a better stirred field.

8. Dimensions of 3D larva models

In Table S1, the dimensions of the 3D models of the larva are given. Notice that length, width and depth are the dimensions of the bounding box, thus length is not the length of a full elongated larva in the case of the anatomical model. The volumes of the water in the Petri dishes for the geometric larva are 8612, 8616, 8623 and 8621 mm^3 for L4 to L1, respectively.

Table S1. Dimensions of the 3D larva models.

Model	Instar	Length [mm]	Width [mm]	Depth [mm]	Volume [mm^3]	Area [mm^2]
Anatomical (µ CT-scan)	L1	1.62	0.60	0.40	0.06	1.62
	L2	2.72	1.01	0.67	0.30	4.57
	L3	4.03	1.49	0.99	0.98	9.99
	L4	6.68	2.46	1.63	4.45	27.47
Geometric (spheroids)	L1	1.75	0.24	0.24	0.05	1.02
	L2	2.94	0.39	0.39	0.24	2.87
	L3	4.35	0.64	0.64	0.93	6.94
	L4	7.21	0.99	0.99	3.68	17.69

9. Dielectric properties

The dielectric properties used in the simulations for 3.6 GHz are given in the Table below.

The material of the Petri dish was polystyrene with a relative permittivity of 2.54 and a conductivity of 0.00047 S/m for all frequencies used.

Table S2. Dielectric properties used in the numerical simulations.

Frequency [GHz]	Material	Relative permittivity		Conductivity [S/m]
3.6	Water	81.92		2.88
	Larva	27.47		2.09
6	Water	77.53		7.53
	Larva	25.36		3.51
12	Water	62.28		23.71
	Larva	19.87		12.99
26	Water	33.52		54.40
	Larva	12.58		15.23
60	Water	13.20		77.28
	Larva	7.42		23.24

10. Rescaling of absorbed power

Simulations are done for a certain incident electric field strength. However, the experiment happens at a different field strength. It is possible to rescale the results of the absorbed power, using:

$$P_{abs,r} = P_{abs}(1\mathrm{V/m})\frac{E_r^2}{(1\mathrm{V/m})^2} \quad (S7)$$

Where E_r is the incident RMS electric field strength we want to rescale to, $P_{abs,r}$ is the absorbed power for the E_r and P_{abs}(1V/m) the absorbed power for an incident RMS electric field strength of 1 V/m. (Thielens, *et al*. 2020)

11. Electric field at different frequencies

In Fig. S8, the simulated electric field strength in water and larva in the Petri dish for different frequencies is shown for a water level of 9.15 mm. It is clear that at higher frequencies the penetration depth in the water becomes smaller and the electric field strength becomes lower. The part of the larva that is near the surface will absorb more EM power than the deeper parts. The relative proportion of instar 1 that is close to the surface is larger than that of instar 4. For 12 GHz and higher frequencies, where the EMFs reach not as deep in the water, the P_{abs} is larger for instar 4 than instar 1 when averaged over volume. The contour of the larva is easily visible in the cross-section for E_2 at 3.6 GHz.

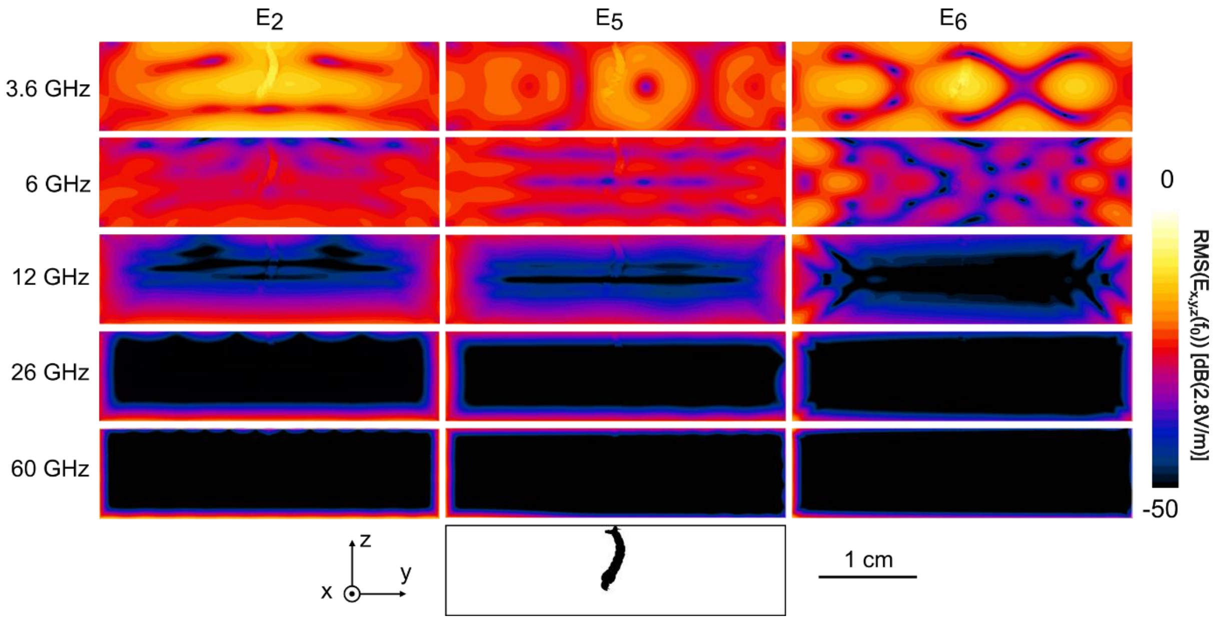

Fig. S8. RMS Electric field strength in a cross-section of the Petri dish wilt larva instar 4 (anatomical model) for different frequencies and 3 different incident plane waves, corresponding to the plane waves of the figure in the paper with an incident RMS electric field strength 1 V/m. On the bottom, the black contour indicates the position of the larva in the surrounding water (white) for all cross-sections.

12. Absorbed power by the water

In Fig. S9, the P_{abs} by the water in the Petri dish is shown for the geometric vertical larvae. When the penetration depth decreases, as seen above, the EM power is mostly absorbed by the edge of the water in the Petri dish. The EMFs will penetrate less into the water and will not reach the insect as much as with a larger penetration depth. At 3.6 GHz, the water absorbs approximately 2×10^{-6} W, while the larva absorbs between 2×10^{-11} W and 4×10^{-9} W. However, the P_{abs}/V of the water is lower than that of the larva, with V the volume. This, and the figure showing the cross-sections (Fig. S8) of the electric field strength, indicate that the insect will have higher dielectric heating than the water at 3.6 GHz. Heat will dissipate from the larva to the water until an equilibrium is reached.

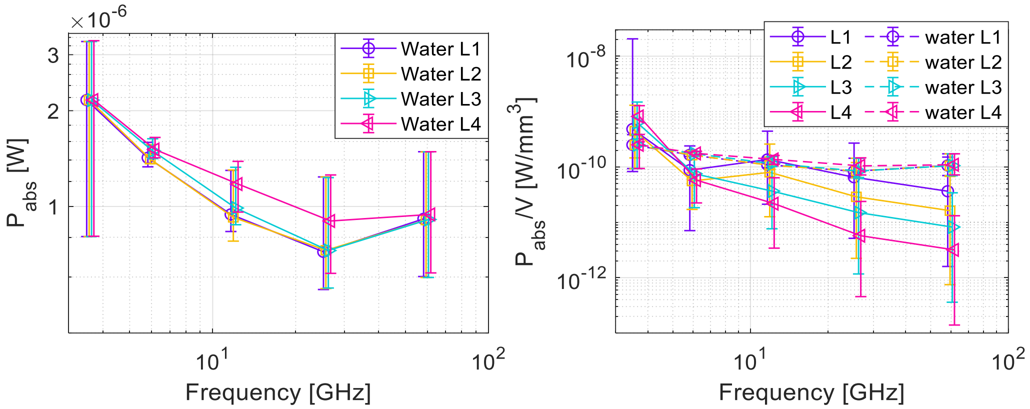

Fig. S9. Absorbed RF-EMF power by the water in the Petri dish (left). Absorbed RF-EMF power by the water and the insect, divided by their volume (right). Whisker indicate the minimum and maximum value found for the 12 plane waves. For incident RMS electric field strength of 1 V/m.

13. Simulation uncertainties

Simulations are an approximation of reality, and uncertainties were considered on the simulations by conducting a sensitivity analysis. The simulations in this analysis were executed for 3.6 GHz, L3 geometric model, at a vertical position (least amount of simulations required), unless indicated differently.

The simulations used in the paper assume a certain water level, dielectric properties of water and insect, and size of the insect. However, no dielectric measurements were executed and the water level was only checked once a day, which gives rise to uncertainties. During the experiment, food for the larvae was added to the water, potentially influencing the dielectric properties of the water. For the dielectric parameters of the larva and of the water, the highest relative difference with the original simulations were taken of the combinations ε ± 10% and σ ± 10%. The influence of the water level was investigated for ± 1 mm. The Insect size was studied between the instars and for ± 3% volume for instar 4.

Furthermore, the simulations had certain settings like voxel size and periods, and a limited set of the incidence plane waves were used. To check whether the twelve plane waves were sufficient, extra simulations were done with plane waves with the same angle of incidence and polarization as for the simulations for the multiple Petri dishes (52 simulations). Additionally, simulations with 50 random incident angles over a sphere and random polarizations of the EMF's were also considered. Furthermore, simulations were done with a voxel size of 2.5 mm instead of 5 mm, as well as with 15 periods instead of 10.

Table S3. Uncertainties on simulation settings and 3D models resulting in uncertainties in absorbed EM power.

Simulation uncertainty	Difference in parameter	Difference in P_{abs}
Water level	± 1 mm	± 32 %
Dielectric properties larva	± 10 %	± 14 %
Dielectric properties water	± 10 %	± 31 %
Voxel size	5 mm - 2.5 mm	± 1 %
Simulated periods	10 - 15	± 1 %
Set incident plane waves	40 extra angles for vertical	± 6 %
Set incident plane waves	40 extra angles for horizontal	± 1 %
Set incident plane waves	50 random incident angles and polarizations	± 6 %
Size	L1 - l4	± 197 %
Size	L3 - l4	± 134 %
Size	L4 ± 3 %	± 1 %
Position	Vertical - horizontal	± 125 %
Type of model	Anatomical – geometrical (L4 vertical)	± 12 %

The effects of the parameters on the simulation results are shown in Table S3. The largest influences are the instar stages and the larva position within the Petri dish. Apart from the previously discussed factors in the paper: position, instar, and model, the largest factor influencing the simulation results was the dielectric properties of the water. The water in the experiments was unpurified, which was also used in the simulations. However, the exact dielectric properties are not known, particularly when food is added the loss factor ε" can deviate ~ 10% (Gadani, *et al*. 2012; Zhao, *et al*. 2019). Furthermore, temperature has an influence on the dielectric properties, but for a change of < 3°C in temperature of the water, less than 10% difference in dielectric properties is expected (Hasted. 1972).

14. Preliminary experiments

To select diets and rearing environment for the experiments, different feeding regimes were investigated and different water containers were tested. Eggs were hydrated and transferred to separate containers one day later, when feeding also started. Tap water was used with 5 mL Tetra AquaSafe (Tetra, Melle, Germany) per 10 L water. A temperature of 28 ± 1°C and relative humidity of 80 ± 5 % were used.

A. Container

The tested containers were the wells of 12-well and 6-well plates (Falcon, Corning Incorporated, Corning, NY, USA), and the 2 mL reaction tubes (Sarstedt, Nümbrecht, Germany), shown in Fig. S10.. During the experiment, the larvae were fed daily with Tetramin. Death rates (DR) were investigated.

Considering that Petri dishes allow for more flexibility, Petri dishes were used in the exposure experiments. With a diameter of 35 mm, the size of the dish is similar to that of the wells in the 6-well plate, which shows to be of suitable size as not many larvae died in the 6 wells, see Table S3. The Petri dishes and wells are both filled with ~ 1 cm of water in height.

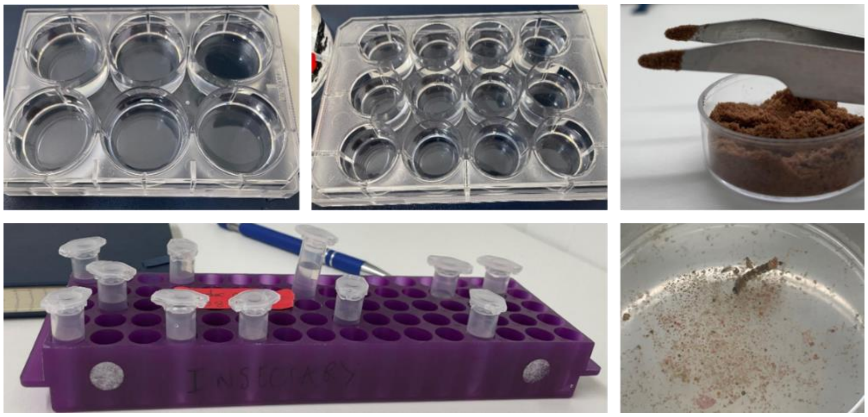

Fig. S10. Containers: 6-well and 12-well plate top left and top middle respectively, 2 mL tubes bottom left. Grinded Tetramin flakes taken with tweezers top right. Tetramin in Petri dish with larva bottom right.

B. Diet

Grinded Tetramin fish food flakes were used as the optimal diet. It was distributed ad libidum, however not so much that it effected the water quality greatly (see Fig. S10). To weaken the mosquitoes, without causing a drastic increase in death rates, dried organic bread yeast (9 g. mixed with tap water to obtain a 40 mL solution) and skimmed milk powder (20 mL, 5 mL and 2 mL mixed with tap water to obtain a 30 mL solution) were used. The larvae were fed one pipette drop of the solution (~ 50 µL), every day until pupation.

In a first preliminary dietary experiment, the containers are tested again, together with a diet of milk (20 mL) or yeast. In Table S4, it was found that the death rates (DR) are similar and it is evident that the 2 mL tube causes a higher death rate than the 6- or 12-well plates.

Table S4. Number of deaths for 24 larvae (for Tetramin) or 12 larvae (for Milk and Yeast) per experiment for different containers and feeding regime.

		2 mL tubes	12 wells	6 wells
Tetramin	Run 1	9	1	3
	Run 2	7	1	0
	Run 3	10	0	0
	Avg DR %	36	3	4
Milk	Run 1	3	2	0
	Run 2	3	0	1
	Run 3	4	1	1
	Avg DR %	28	8	6
Yeast	Run 1	7	1	1
	Run 2	4	2	0
	Run 3	4	0	1
	Avg DR %	42	8	6

From the death rate, it is not clear whether the 20 mL milk will weaken the larvae compared to a diet of Tetramin. To find the right concentrations for a weakened group during the exposure experiments, more experiments were conducted, all in the Petri dishes discussed above. First the number of deaths are again shown in Table S5 for the 5 mL and 2 mL milk solutions. The death rate seems unaffected by the concentration (see also Table S3 for the 20 mL).

Table S5. Number of deaths for 12 larvae per experiment for concentrations of skimmed milk powder.

	2 mL milk	5 mL milk
Run 1	2	1
Run 2	0	2
Run 3	1	0
Avg DR %	8	8

Table S6. Wing lengths for different feeding regimes, run 2.

**Sex**	**Diet**	**Sample size**	**Mean (µm)**	**Std. Dev. (µm)**
Female	2 mL milk	9	2511.34	142.84
	5 mL milk	15	2829.51	156.51
	Tetramin	14	2962.32	83.28
Male	2 mL milk	15	2055.32	88.16
	5 mL milk	15	2264.22	128.29
	Tetramin	20	2264.55	053.68

Apart from death rate, the wing length of the emerged adult were used to check fitness. In Table S6, the wing lengths are given for the female and male adults and for a feeding regime of 2 mL milk, 5 mL milk and Tetramin.

The unpaired t-test was used to determine whether the diet had an influence on the wing length, when the F-test indicated unequal means, the unpaired t-test for unequal variance was used (Welch's t-test). The results are given in Table S7. The t-test shows that for males, the diet does not affect the wing length when the 5 mL-milk and Tetramin are compared. All other wing lengths seemed to be affected by the feeding regime, with the greatest effect between 2 mL and Tetramin.

Table S7. Results of unpaired t-test for wing lengths for different diets of run 2. When degrees of freedom is a decimal number, the unpaired t-test for unequal variance (Welch's t-test) was used because F-test indicated unequal means.

**Sex**	**Diets**	**t-value**	**Df**	**p-value**
Female	5 mL – Tetramin	-2.88	21.64	8.81E-03
Female	2 mL – 5 mL	-4.97	22	5.06E-05
Female	2 mL - Tetramin	-9.61	21	3.87E-09
Male	5 mL – Tetramin	-0.01	17.69	9.93E-01
Male	2mL – 5 mL	-5.20	28	1.62E-05
Male	2 mL - Tetramin	-8.13	21.63	5.10E-08

For strong larvae, Tetramin was used, for weakened larvae, 2 mL milk was used in the exposure experiment. Two more runs were executed on the 2 mL milk and Tetramin to verify the results. These wing lengths are shown in Fig. S11.

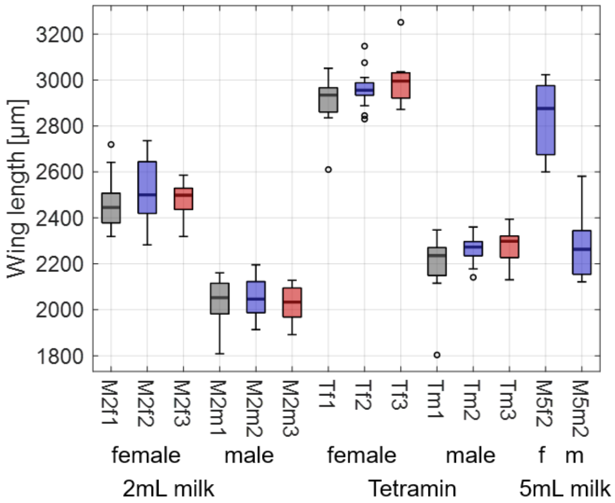

Fig. S11. Boxplot for the wing lengths of the 3 different runs for female (f) and male (m) for different diets of 2mL Milk (M2) and Tetramin (T) and for 1 run for 5mL Milk (M5).

15. Day of Event per experiment run

In Fig. S12, the raw data of the moment of event (pupation or adult emergence) is shown. The amount of pupations and adult emergencies are shown per day per experiment run. For the LC experiments, 8 runs were executed. Due to high amounts of unexpected deaths for the larvae fed with milk, 3 of the runs were discarded with the believe the milk used in these runs was expired. In this way, all experiment type had 5 runs. At the end of HC1M, 29 larva were unexpectedly not yet pupated, these were not considered as deaths. HE5M shows a later development than other HEM runs, no reason was found and the results were not omitted. Table S8 shows the mean day of adult emergence and pupation per group.

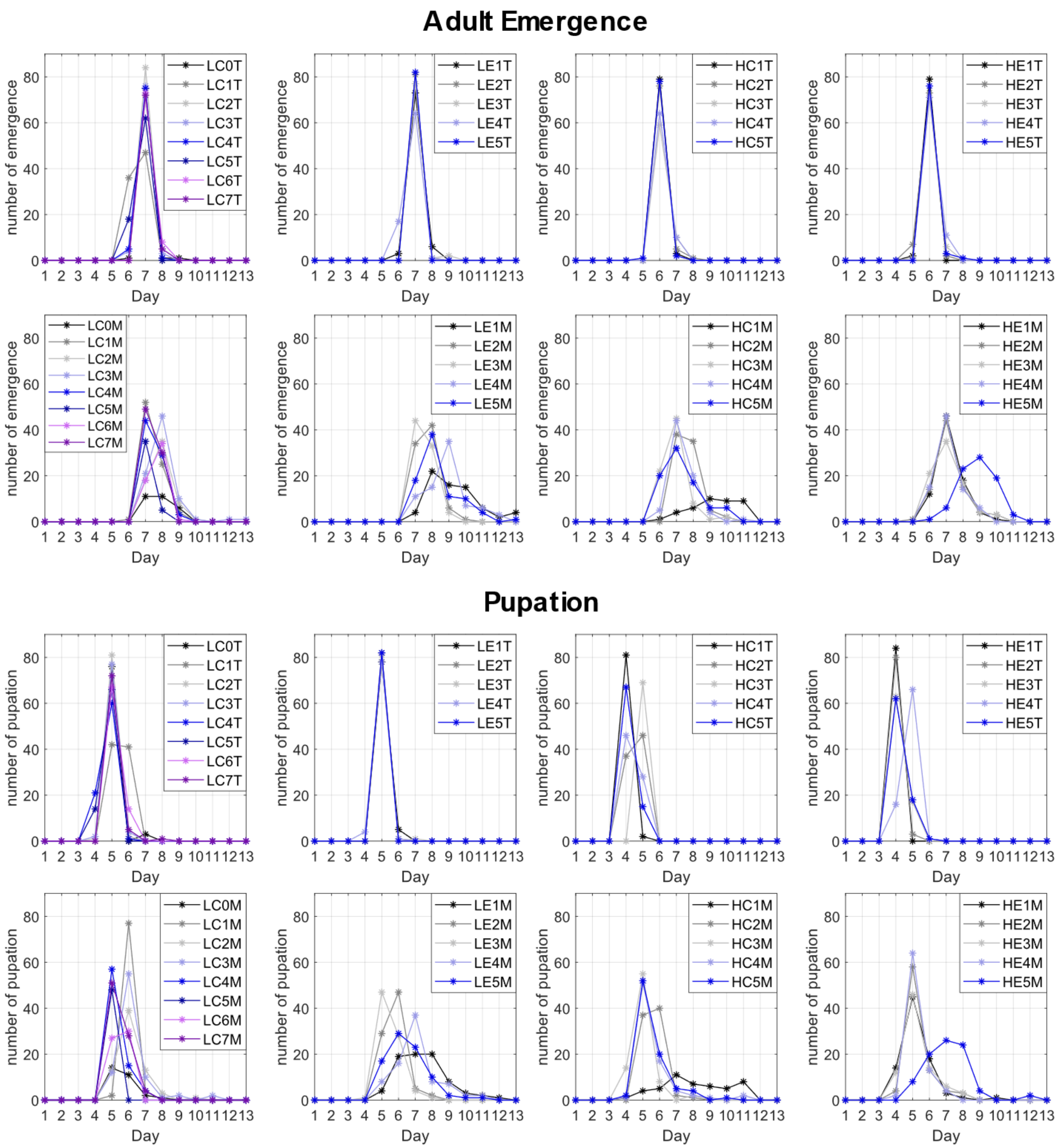

Fig. S12. Amount of emerged adults or pupated mosquitoes per day per experiment run. T: Tetramin, M: Milk.

Table S8. Mean of the day of Adult emergence and pupation per group, with standard deviation (sd) and standard error (se).

		Adult emergence [day]			Pupation [day]		
**Run type**	**Diet**	**Mean**	**sd**	**se**	**Mean**	**sd**	**se**
HC	T	6.08	0.29	0.01	4.41	0.49	0.02
HC	M	7.53	1.16	0.06	5.77	1.43	0.07
LC	T	6.91	0.35	0.02	5.08	0.45	0.02
LC	M	7.61	0.74	0.04	5.82	0.81	0.04
HE	T	6.03	0.30	0.01	4.26	0.45	0.02
HE	M	7.51	1.13	0.06	5.63	1.19	0.06
LE	T	6.98	0.30	0.01	5.01	0.19	0.01
LE	M	8.26	1.22	0.06	6.42	1.32	0.07

16. Wing Length per experiment run

In Table S9, the mean wing length of the different groups of the different experiments is shown, together with the standard deviation (sd) and the standard error (se). In Table S10 and Table S11, the mean wing length and standard deviation are given per run.

Table S9. Mean wing lengths, amount of samples, standard deviation (sd) and standard error (se) for the different experiment groups.

Type	Diet	Sex	Mean wing length [µm]	n	sd [µm]	se [µm]
HC	M	f	2547.39	155	144.29	11.59
HC	M	m	2042.35	197	112.18	7.99
HC	T	f	2905.76	169	91.58	7.04
HC	T	m	2237.67	202	66.20	4.66
HE	M	f	2517.07	192	101.32	7.31
HE	M	m	2055.11	200	82.48	5.83
HE	T	f	2916.97	192	81.21	5.86
HE	T	m	2246.82	185	58.19	4.28
LC	M	f	2606.61	130	119.47	10.48
LC	M	m	2118.54	169	79.98	6.15
LC	T	f	2992.58	153	87.84	7.10
LC	T	m	2294.68	176	63.44	4.78
LE	M	f	2562.48	204	129.89	9.09
LE	M	m	2083.18	250	117.29	7.42
LE	T	f	2990.74	228	86.26	5.71
LE	T	m	2301.30	245	65.59	4.19

Table S10. Mean Wing length and standard deviation (sd) for the different runs of the Milk fed mosquitoes.

Run	n females	Mean [µm]	sd [µm]	n males	Mean [µm]	sd [µm]
HC1	13	2270.70	133.49	26	1872.29	86.36
HC2	40	2524.99	124.35	39	2031.67	89.44
HC3	40	2586.78	92.11	38	2049.82	73.95
HC4	29	2597.93	97.76	46	2102.21	98.03
HC5	33	2591.37	134.10	48	2079.85	86.36
HE1	42	2546.30	98.74	39	2066.16	63.51
HE2	33	2482.62	87.25	47	2046.18	69.32
HE3	35	2510.93	102.57	44	2075.78	99.71
HE4	39	2554.58	104.38	35	2064.37	59.40
HE5	43	2485.94	94.22	35	2019.53	102.51
LC1	41	2614.91	95.07	50	2129.35	76.80
LC2	25	2579.20	116.24	33	2106.31	86.99
LC3	33	2671.75	93.76	45	2115.67	79.80
LC4	31	2548.39	142.30	41	2118.33	79.38
LE1	66	2554.20	126.97	84	2070.73	105.37
LE2	41	2580.38	94.09	37	2096.78	83.96
LE3	30	2657.93	107.13	50	2184.31	74.42
LE4	31	2470.89	117.06	36	1983.10	100.37
LE5	36	2556.59	144.10	43	2062.00	130.51

Table S11. Mean Wing length and standard deviation (sd) for the different runs of the Tetramin fed mosquitoes.

run	n females	Mean [µm]	sd [µm]	n males	Mean [µm]	sd [µm]
HC1	35	2899.6	102.4	36	2244.74	53.90
HC2	43	2857.2	84.4	35	2173.52	59.69
HC3	30	2916.1	82.5	37	2227.29	74.97
HC4	31	2925.1	71.2	42	2256.40	46.42
HC5	30	2952.3	87.5	52	2268.23	55.02
HE1	39	2949.5	80.8	39	2268.78	63.00
HE2	38	2909.7	73.1	38	2262.31	58.24
HE3	39	2952.9	69.8	31	2252.61	52.04
HE4	39	2883.3	83.6	36	2218.79	46.79
HE5	37	2887.9	73.4	41	2231.81	55.32
LC1	36	2951.2	84.4	49	2281.28	47.25
LC2	40	2982.2	58.2	41	2297.96	60.88
LC3	39	3051.1	97.2	40	2343.73	55.65
LC4	38	2982.5	78.7	46	2263.39	62.82
LE1	75	2992.3	92.4	88	2298.38	65.34
LE2	47	3005.2	78.0	33	2278.22	70.60
LE3	28	3003.4	84.3	51	2308.73	65.36
LE4	46	2976.0	81.4	27	2317.26	55.91
LE5	32	2975.7	90.8	46	2305.85	65.82

17. LMM Wing length female and male

The female and male *Ae. aegypti* wing lengths were measured and a linear mixed effect model (LMM) was made for both. For the female model, as well as the male model, the results are shown in Table S12 and Table S13, for different baselines. The first results in the table are for baseline LC Tetramin, followed by LC Milk, HC Tetramin and HC Milk.

It is known that female *Ae. Aegypti* are larger than the males, in (De Majo, *et al*. 2021) it was shown a significant ($p<0.05$) interaction between sex and temperature exist. However, as finding the effect of sex is not the main goal and to reduce complexity, the sexes were taken separate. The effects found in the male LMM are similar to those of the female LMM.

18. Difference between runs

Every experiment type was repeated at least 5 times. For some of the experiments type, the wing lengths of only 4 of the 5 runs were checked. To check the difference between the runs for the wing length, a Kruskal-Wallis test (for Barlett's test $p < 0.05$) or ANOVA test was performed and shown in Table S14. Between the runs of most experimental conditions, a difference in runs is found, however the difference is considered relatively small in most cases, see also Table S10 and Table S11.

The difference between the runs for the development timing is also measured with a Kruskal-Wallis test. In all groups Pr(>Chisq) < 10^{-10}, except for the adult emergence of Tetramin HE (Chisq = 23.67, Pr(>Chisq) = 9.30×10^{-5}), adult emergence of Tetramin HC (Chisq = 13.45, Pr(>Chisq) = 9.30×10^{-3}) and pupation of Tetramin LE (Chisq = 13.54, Pr(>Chisq) = 8.90×10^{-3}), which are still significantly different for a significance level of 0.05. The difference between runs are also clear from Fig. S12.

Table S12. LMM results wing length for the female model.

Effect	Estimate	Std.error	t-value	df	p-value	Sign.
(Intercept) LC T	2992.23	21.08	141.93	16.89	2.01e-27	***
Type HC	-82.14	28.42	-2.89	17.20	1.01e-02	*
Type HE	-75.63	28.28	-2.67	16.88	1.61e-02	*
Type LE	2.18	28.18	0.08	16.62	9.39e-01	
Food M	-386.16	12.14	-31.82	1402.27	1.02e-167	***
Type HC:food M	15.41	16.67	0.92	1405.68	3.56e-01	
Type HE:food M	-13.49	15.95	-0.85	1401.45	3.98e-01	
Type LE:food M	-45.02	15.59	-2.89	1401.87	3.94e-03	**
Sd (intercept)	38.86	Na	Na	Na	Na	
Sd observation	101.22	Na	Na	Na	Na	
(Intercept) LC M	2606.07	21.38	121.87	17.87	1.53e-27	***
Type HC	-66.73	28.79	-2.32	18.10	3.24e-02	*
Type HE	-89.12	28.51	-3.13	17.43	6.01e-03	**
Type LE	-42.84	28.48	-1.50	17.36	1.51e-01	
Food T	386.16	12.14	31.82	1402.27	1.02e-167	***
Type HC:food T	-15.41	16.67	-0.92	1405.68	3.56e-01	
Type HE:food T	13.49	15.95	0.85	1401.45	3.98e-01	
Type LE:food T	45.02	15.59	2.89	1401.87	3.94e-03	**
Sd (intercept)	38.86	Na	Na	Na	Na	
Sd observation	101.22	Na	Na	Na	Na	
(Intercept) HC T	2910.09	19.06	152.72	17.60	6.13e-29	***
Type LC	82.14	28.42	2.89	17.20	1.01e-02	*
Type HE	6.50	26.80	0.24	17.23	8.11e-01	
Type LE	84.32	26.69	3.16	16.94	5.75e-03	**
Food M	-370.75	11.43	-32.42	1408.89	9.23e-173	***
Type LC:food M	-15.41	16.67	-0.92	1405.68	3.56e-01	
Type HE:food M	-28.90	15.42	-1.87	1405.46	6.11e-02	.
Type LE:food M	-60.43	15.05	-4.02	1406.01	6.25e-05	***
Sd (intercept)	38.86	Na	Na	Na	Na	
Sd observation	101.22	Na	Na	Na	N	
(Intercept) HC M	2539.34	19.28	131.74	18.39	8.07e-29	***
Type LC	66.73	28.79	2.32	18.10	3.24e-02	*
Type HE	-22.40	26.96	-0.83	17.63	4.17e-01	
Type LE	23.89	26.94	0.89	17.55	3.87e-01	
Food T	370.75	11.43	32.42	1408.89	9.23e-173	***
Type LC:food T	15.41	16.67	0.92	1405.68	3.56e-01	
Type HE:food T	28.90	15.42	1.87	1405.46	6.11e-02	.
Type LE:food T	60.43	15.05	4.02	1406.01	6.25e-05	***
Sd (intercept)	38.86	Na	Na	Na	Na	
Sd observation	101.22	Na	Na	Na	Na	

Table S13. LMM results wing length for the male model.

Effect	Estimate	Std.error	t-value	df	p-value	Sign.
(Intercept) LC T	2295.29	16.73	137.23	16.86	3.79e-27	***
Type HC	-61.97	22.50	-2.75	17.05	1.35e-02	*
Type HE	-47.77	22.56	-2.12	17.24	4.91e-02	*
Type LE	2.32	22.42	0.10	16.79	9.19e-01	
Food M	-177.33	8.66	-20.47	1601.80	6.47e-83	***
Type HC:food M	-20.51	11.83	-1.73	1602.06	8.32e-02	.
Type HE:food M	-16.13	11.94	-1.35	1602.33	1.77e-01	
Type LE:food M	-39.07	11.28	-3.46	1601.77	5.49e-04	***
Sd (intercept)	31.18	Na	Na	Na	Na	
Sd observation	80.29	Na	Na	Na	Na	
(Intercept) LC M	2117.96	16.78	126.22	17.08	8.25e-27	***
Type HC	-82.48	22.57	-3.65	17.25	1.92e-03	**
Type HE	-63.90	22.55	-2.83	17.19	1.14e-02	*
Type LE	-36.75	22.43	-1.64	16.82	1.20e-01	
Food T	177.33	8.66	20.47	1601.80	6.47e-83	***
Type HC:food T	20.51	11.83	1.73	1602.06	8.32e-02	.
Type HE:food T	16.13	11.94	1.35	1602.33	1.77e-01	
Type LE:food T	39.07	11.28	3.46	1601.77	5.49e-04	***
Sd (intercept)	31.18	Na	Na	Na	Na	
Sd observation	80.29	Na	Na	Na	Na	
(Intercept) HC T	2233.32	15.06	148.34	17.29	2.69e-28	***
Type LC	61.97	22.50	2.75	17.05	1.35e-02	*
Type HE	14.20	21.36	0.67	17.50	5.15e-01	
Type LE	64.29	21.20	3.03	16.99	7.52e-03	**
Food M	-197.84	8.06	-24.55	1602.35	3.21e-113	***
Type LC:food M	20.51	11.83	1.73	1602.06	8.32e-02	.
Type HE:food M	4.38	11.51	0.38	1602.64	7.04e-01	
Type LE:food M	-18.56	10.83	-1.71	1602.08	8.67e-02	.
Sd (intercept)	31.18	Na	Na	Na	Na	
Sd observation	80.29	Na	Na	Na	Na	
(Intercept) HC M	2035.48	15.09	134.87	17.46	8.50e-28	***
Type LC	82.48	22.57	3.65	17.25	1.92e-03	*
Type HE	18.58	21.32	0.87	17.39	3.95e-01	
Type LE	45.74	21.19	2.16	16.98	4.56e-02	
Food T	197.84	8.06	24.55	1602.35	3.21e-113	***
Type LC:food T	-20.51	11.83	-1.73	1602.06	8.32e-02	
Type HE:food T	-4.38	11.51	-0.38	1602.64	7.04e-01	.
Type LE:food T	18.56	10.83	1.71	1602.08	8.67e-02	***
Sd (intercept)	31.18	Na	Na	Na	Na	
Sd observation	80.29	Na	Na	Na	Na	

Table S14. Comparison between runs for wing length. For the groups with p < 0.05 for Barlett's test, the Kruskal-Wallis test is used, else ANOVA was used.

			**Barlets**			**Anova/kruskal-wallis**		
**Type**	**Diet**	**Sex**	**Ksq**	**df**	**p**	**F/Chisq**	**df**	**p**
HC	M	f	7.31	4	0.12	22.89	4	<0.001
HE	M	f	1.38	4	0.85	4.52	4	0.002
LC	M	f	7.76	3	0.05	7.11	3	<0.001
LE	M	f	7.78	4	0.1	9.57	4	<0.001
HC	T	f	4.32	4	0.36	6.09	4	<0.001
HE	T	f	1.74	4	0.78	7.25	4	<0.001
LC	T	f	9.86	3	0.02	34.6	3	<0.001
LE	T	f	2.07	4	0.72	1.07	4	0.372
HC	M	m	3.16	4	0.53	32.23	4	<0.001
HE	M	m	19.76	4	<0.001	7.9267	4	0.094
LC	M	m	0.63	3	0.89	0.58	3	0.631
LE	M	m	16.47	4	<0.001	69.58	4	<0.001
HC	T	m	9.78	4	0.04	48.16	4	<0.001
HE	T	m	3.53	4	0.47	5.38	4	<0.001
LC	T	m	4.26	3	0.24	15.58	3	<0.001
LE	T	m	1.53	4	0.82	1.7	4	0.150

19. Mortality rate

For milk, the mean mortality rates ± standard error over the 5 replicates are 9.7 ± 2.6 %, 5.1 ± 1.1 %, 9.0 ± 3.4 % and 6.3 ± 2.1 % for HC, HE, LC and LE, respectively. For Tetramin, the mean mortality rates ± standard error over the 5 replicates are 8.0 ± 3.3 %, 3.8 ± 1.0 %, 3.8 ± 1.2 % and 3.1 ± 0.5 % for HC, HE, LC and LE, respectively.

20. Wing length Asymmetry

In Table S15, asymmetry is expressed in absolute difference between left and right wing length.

21. Pairwise Wilcoxon rank sum test on development time

A pairwise Wilcoxon rank sum test was done on the development time of the different exposure conditions. The development time was taken as day of development for every larva. The results are given in Table S16. A clear difference is found for diet. Additionally, a difference in development time between exposure conditions is found with a significance level 0.01, except for HE and HC. For HE and HC, no significant difference is seen when fed with milk. there is a difference at pupation between development time for HC and HE when fed with Tetramin, however, this changes for adult emergence.

Table S15. Wing length asymmetry: absolute difference between left and right wing length.

**Group**	**Sex**	**Diet**	**mean |L-R| [μm]**	**95 % C.I. [μm]**	**sd [μm]**	**n**
HC	f	M	23.87	(0.77,62.79)	17.65	53
HC	f	T	24.84	(1.05,52.18)	16.37	50
HC	m	M	18.81	(1.22,46.47)	13.08	56
HC	m	T	20.45	(1.84,49.35)	13.79	55
HE	f	M	26.14	(5.72,67.95)	17.05	33
HE	f	T	32.08	(9.93,61.90)	16.26	19
HE	m	M	14.10	(1.44,31.55)	8.83	30
HE	m	T	14.85	(0.55,52.13)	15.56	19
LC	f	M	22.33	(0.77,50.93)	16.49	30
LC	f	T	19.32	(0.54,49.31)	14.39	31
LC	m	M	21.54	(1.91,44.91)	13.66	30
LC	m	T	19.92	(0.82,52.87)	14.32	30
LE	f	M	19.66	(0.87,60.58)	14.44	28
LE	f	T	24.78	(2.54,52.20)	14.73	29
LE	m	M	19.17	(2.03,44.63)	13.32	27
LE	m	T	18.66	(1.51,60.42)	15.19	29

Table S16. Pairwise Wilcoxon rank sum test on development time.

**Group 1**		**Group 2**		**Pupation**	**Adult emergence**
**Exposure conditions**	**diet**	**Exposure conditions**	**diet**	**p-value**	**p-value**
LC	M	LE	M	<0.001	<0.001
LC	M	HC	M	<0.001	0.002
LC	M	HE	M	<0.001	0.002
LE	M	HC	M	<0.001	<0.001
LE	M	HE	M	<0.001	<0.001
HC	M	HE	M	**0.381**	**0.873**
LC	T	LE	T	0.004	0.003
LC	T	HC	T	<0.001	<0.001
LC	T	HE	T	<0.001	<0.001
LE	T	HC	T	<0.001	<0.001
LE	T	HE	T	<0.001	<0.001
HC	T	HE	T	<0.001	**0.027**
LC	M	LC	T	<0.001	<0.001
LE	M	LE	T	<0.001	<0.001
HC	M	HC	T	<0.001	<0.001
HE	M	HE	T	<0.001	<0.001

22. Proportional Hazard Assumption

To use the logrank test for the time to pupation or time to adult emergence, the assumption of proportional hazards needs to be met. For every two groups, the proportional hazards assumption was checked pairwise according to the Schoenfeld residuals (Table S17). For a significance level of 0.05, the Tetramin-fed HC and HE do not have proportional hazards for their pupation.

Table S17. Proportional hazard check between two groups using Schoenfeld residuals.

Event	Diet	Group 1	Group 2	Chisq	p
Pupation	M	LC	LE	0.53	0.47
Pupation	M	HC	HE	2.86	0.091
Pupation	T	LC	LE	3.22	0.073
Pupation	T	HC	HE	10.1	0.002
Adult em.	M	LC	LE	3.13	0.077
Adult em.	M	HC	HE	0.56	0.45
Adult em.	T	LC	LE	0.05	0.83
Adult em.	T	HC	HE	0.89	0.35

23. Sex ratios

The male *Ae. aegypti* have a shorter development time compared to females for larva instar 1 to pupation and from larva instar 1 to adult emergence (Souza, *et al*. 2019). The sex of different groups were examined to see if the sex ratios were similar and uneven ratios did not cause differences in development time. Sex ratio is defined as the amount of males emerged as adults over the total amount of emerged adults and is shown in Table S18 for four different runs of different experiment type (LC, LE, HC and HE) and feeding regime (T and M). The number of the runs in the table are not necessarily the same as the numbers of the runs in the paragraphs above. Death larvae and pupae were not sexed and thus not included in this table, damaged (due to handling) or escaped adults were not included either.

More males than females were recorded, as expected (Souza, *et al*. 2019), except for the HE Tetramin group, were females dominated. The differences in sex ratio is considered small and neglected in the analysis of the development.

Table S18. The sex ratios for different runs of different experiments (group).

Group	Run1	Run2	Run3	Run4	Total male	Total female	Total sex ratio
LC T	59%	52%	51%	54%	180	154	54%
LC M	55%	58%	59%	57%	176	132	57%
LE T	42%	65%	38%	60%	163	155	51%
LE M	47%	63%	55%	56%	172	140	55%
HC T	46%	56%	57%	63%	169	135	56%
HC M	50%	49%	62%	59%	173	142	55%
HE T	38%	50%	45%	51%	134	155	46%
HE M	48%	58%	56%	50%	170	150	53%

Supplementary References

Arnaut, L. R. (2003). Statistics of the quality factor of a rectangular reverberation chamber. *IEEE Transactions on electromagnetic compatibility*, 45(1), 61-76.

Bar, A., & Andrew, J. (2013). Morphology and morphometry of Aedes aegypti larvae. *Annual review and Research in Biology*, 3(1), 1-21.

Besnier, P., Lemoine, C., & Sol, J. (2015, August). Various estimations of composite Q-factor with antennas in a reverberation chamber. *In 2015 IEEE International Symposium on Electromagnetic Compatibility (EMC)* (pp. 1223-1227). IEEE.

Chen, X., Kildal, P. S., & Lai, S. H. (2011). Estimation of average Rician K-factor and average mode bandwidth in loaded reverberation chamber. *IEEE Antennas and Wireless Propagation Letters*, *10*, 1437-1440.

De Majo, M. S., Zanotti, G., Gimenez, J. O., Campos, R. E., & Fischer, S. (2021). Comparative study on the thermal performance of three Aedes aegypti (Diptera: Culicidae) populations from Argentina. *Journal of Medical Entomology*, 58(4), 1733-1739.

Gadani, D. H., Rana, V. A., Bhatnagar, S. P., Prajapati, A. N., & Vyas, A. D. (2012). Effect of salinity on the dielectric properties of water. *Indian Journal of Pure & Applied Physics*, 50, 405-410.

Hasted, J. B. (1972). Liquid water: Dielectric properties. In *The Physics and Physical Chemistry of Water* (pp. 255-309). Boston, MA: Springer New York.

Holloway, C. L., Hill, D. A., Ladbury, J. M., & Koepke, G. (2006). Requirements for an effective reverberation chamber: Unloaded or loaded. *IEEE Transactions on Electromagnetic Compatibility*, 48(1), 187-194.

IEC 61000-4-21. (2011). Electromagnetic compatibility (EMC)–Part 4–21: Testing and measurement techniques–Reverberation chamber test methods.

Krauthauser, H. G. (2012). Number of samples required to meet a field inhomogeneity limit with given confidence in reverberation chambers. *IEEE transactions on electromagnetic compatibility*, 54(5), 968-975.

Lehnebach, R., Campioli, M., Gričar, J., Prislan, P., Mariën, B., Beeckman, H., & Van den Bulcke, J. (2021). High-resolution X-ray computed tomography: a new workflow for the analysis of xylogenesis and intra-seasonal wood biomass production. *Frontiers in Plant Science*, 12, 698640.

Lemoine, C., Besnier, P., & Drissi, M. H. (2007). Investigation of reverberation chamber measurements through high-power goodness-of-fit tests. *IEEE Transactions on Electromagnetic Compatibility*, 49(4), 745-755.

Senic, D., Remley, K. A., Wang, C. M. J., Williams, D. F., Holloway, C. L., Ribeiro, D. C., & Kirk, A. T. (2016). Estimating and reducing uncertainty in reverberation-chamber characterization at millimeter-wave frequencies. *IEEE Transactions on Antennas and Propagation*, *64*(7), 3130-3140.

Souza, R. S., Virginio, F., Riback, T. I. S., Suesdek, L., Barufi, J. B., & Genta, F. A. (2019). Microorganism-based larval diets affect mosquito development, size and nutritional reserves in the yellow fever mosquito Aedes aegypti (Diptera: Culicidae). *Frontiers in physiology*, 10, 152.

Thielens, A., Greco, M. K., Verloock, L., Martens, L., & Joseph, W. (2020). Radio-frequency electromagnetic field exposure of western honey bees. *Scientific Reports*, 10(1), 461.

Yousaf, J., Nah, W., Hussein, M. I., Yang, J. G., Altaf, A., & Elahi, M. (2020). Characterization of reverberation chamber-a comprehensive review. *IEEE Access*, 8, 226591-226608.

Zhao, K., Liu, Y., & Zhang, Q. (2019). Dielectric behavior of adulterated milk with urea and water. *Journal of Molecular Liquids*, 273, 37-44.
